# Zebrafish Tric-b is required for skeletal development and bone cells differentiation

**DOI:** 10.3389/fendo.2023.1002914

**Published:** 2023-01-23

**Authors:** Francesca Tonelli, Laura Leoni, Valentina Daponte, Roberta Gioia, Silvia Cotti, Imke A. K. Fiedler, Daria Larianova, Andy Willaert, Paul J. Coucke, Simona Villani, Björn Busse, Roberta Besio, Antonio Rossi, P. Eckhard Witten, Antonella Forlino

**Affiliations:** ^1^ Department of Molecular Medicine, Biochemistry Unit, University of Pavia, Pavia, Italy; ^2^ Department of Biology, Ghent University, Ghent, Belgium; ^3^ Department of Osteology and Biomechanics, University Medical Center Hamburg-Eppendorf, Hamburg, Germany; ^4^ Department of Biomolecular Medicine, Center of Medical Genetics, Ghent University-University Hospital, Ghent, Belgium; ^5^ Department of Public Health and Experimental and Forensic Medicine, Unit of Biostatistics and Clinical Epidemiology, University of Pavia, Pavia, Italy

**Keywords:** zebrafish, TRIC-B, osteogenesis imperfecta, bone, fin regeneration, collagen, endoplasmic reticulum

## Abstract

**Introduction:**

Trimeric intracellular potassium channels TRIC-A and -B are endoplasmic reticulum (ER) integral membrane proteins, involved in the regulation of calcium release mediated by ryanodine (RyRs) and inositol 1,4,5-trisphosphate (IP_3_Rs) receptors, respectively. While TRIC-A is mainly expressed in excitable cells, TRIC-B is ubiquitously distributed at moderate level. TRIC-B deficiency causes a dysregulation of calcium flux from the ER, which impacts on multiple collagen specific chaperones and modifying enzymatic activity, leading to a rare form of osteogenesis imperfecta (OI Type XIV). The relevance of TRIC-B on cell homeostasis and the molecular mechanism behind the disease are still unknown.

**Results:**

In this study, we exploited zebrafish to elucidate the role of TRIC-B in skeletal tissue. We demonstrated, for the first time, that *tmem38a* and *tmem38b* genes encoding Tric-a and -b, respectively are expressed at early developmental stages in zebrafish, but only the latter has a maternal expression. Two zebrafish mutants for *tmem38b* were generated by CRISPR/Cas9, one carrying an out of frame mutation introducing a premature stop codon (*tmem38b^-/-^*) and one with an in frame deletion that removes the highly conserved KEV domain (*tmem38b^Δ120-7/Δ120-7^*). In both models collagen type I is under-modified and partially intracellularly retained in the endoplasmic reticulum, as described in individuals affected by OI type XIV. *Tmem38b^-/-^* showed a mild skeletal phenotype at the late larval and juvenile stages of development whereas *tmem38b^Δ120-7/Δ120-7^* bone outcome was limited to a reduced vertebral length at 21 dpf. A caudal fin regeneration study pointed towards impaired activity of osteoblasts and osteoclasts associated with mineralization impairment.

**Discussion:**

Our data support the requirement of Tric-b during early development and for bone cell differentiation.

## 1 Introduction

The trimeric cation channels, TRICs, are responsible for the transport of K^+^ ions across the endoplasmic or sarcoplasmic reticulum (ER/SR) membranes where they act as counter-ions to allow electroneutral Ca^2+^ exit from the ER/SR lumen to the cytosol. The mammalian family of TRICs is composed by TRIC-A and TRIC-B subtypes, encoded by *TMEM38A* and *TMEM38B*, respectively. TRIC-A mediated Ca^2+^ release throughout coupling with ryanodine receptors (RyRs) is particularly relevant in excitable cells, especially in striated muscle and brain (1), while TRIC-B, synchronizing with inositol trisphosphate receptors (IP_3_Rs), ubiquitously mediates Ca^2+^ release (2).

Calcium plays important roles both as cofactor and stabilizing ion for several proteins with enzymatic and/or chaperone functions and as second messenger in various signal transduction pathways, thus its homeostasis affects multiple cell functions, including contraction and relaxation, motility, metabolism, protein synthesis, modification and folding, secretion, cell-cycle progression, apoptosis and gene expression (3, 4).

The impairment in RyRs- and IP_3_Rs- mediated Ca^2+^ release observed in TRIC-A and TRIC-B knock-out mouse models, respectively, supports the tissue specific role of the channels in modulating calcium signaling. TRIC-A knock-out mice show reduced or irregular muscle contractile responses and develop hypertension (5, 6), while TRIC-B knock-out mice suffer from pulmonary dysfunction and die perinatally due to an insufficient alveoli surfactant production (7). Interestingly, TRIC-B knock-out mice reveal also skeletal abnormalities, such as reduced body size and impaired ossification associated with insufficient collagen matrix production (8).

A skeletal phenotype is similarly described in humans with loss of function mutations in *TMEM38B*, which are affected by a recessive form of osteogenesis imperfecta (OI), classified as OI type XIV (OMIM 615066) (9). They are characterized, similarly to the most OI individuals with dominant and other recessive forms (10), by wide phenotypical variability, ranging from asymptomatic to severe, with different degrees of bone deformities, low bone mass, mild to recurrent fractures, growth retardation and short stature (11). The primary fibroblasts and osteoblasts isolated from affected individuals display a reduced level of helical lysyl hydroxylation, suggesting a dysfunctional activity of lysyl hydroxylase 1 (LH1) that is a calcium dependent enzyme. LH1 is necessary for proper lysine hydroxylation in the collagen type I helical domain and formation of stable intermolecular cross-linking (12). Furthermore, hydroxylysines are substrates for collagen specific ER O-linked glycosylation process (13, 14). In OI type XIV individuals this results in the synthesis of under-modified collagen type I (15, 16), which is susceptible to cell retention causing ER stress and increased degradation. Furthermore, abnormally secreted molecules are not properly incorporated in the extracellular matrix fibers (17). Also, osteoblasts from TRIC-B knock-out mice present enlarged ER cisternae due to intracellular collagen retention (8).

*In vitro* long term cultured primary human OI type XIV osteoblasts display reduced expression of early markers of differentiation such as *RUNX2* and *SP7* and increased expression of the later markers *BGLAP* and *OPN*, which inhibit crystal growth (16, 18). Osteoclasts are also impaired, since their number and activity are reduced (16). In immortalized human Foetal Osteoblasts (hFOB) knock-out for *TMEM38B* a decreased proliferation and mineralization have been recently demonstrated (19).

In the last decade, zebrafish proved to be a very reliable animal model to reproduce human common and heritable disorders, including skeletal diseases. Its high reproductive rate, larvae transparency and small size together with the ease of manipulation of its genome made zebrafish a high throughput and low-cost model to understand the molecular basis of human diseases as well as to identify new targets and to test innovative pharmacological approaches. The development of high-resolution imaging techniques further strength the use of teleost in bone research (20).

Zebrafish can regenerate several organs and tissues including bone and indeed fin ray regeneration represents a powerful tool to investigate bone formation in adult animals.

In this study we mapped for the first time the spatial and temporal expression of *tmem38a* and *tmem38b* during zebrafish early developmental stages and their expression in adult tissues. By CRISPR/Cas9 targeting of *tmem38b* in zebrafish we proved the relevance of Tric-b for skeletal development and collagen biosynthesis. Using a caudal fin regeneration assay we demonstrated that lack of *tmem38b* affects both osteoblasts and osteoclasts activity.

## 2 Materials and methods

### 2.1 Zebrafish husbandry and ethical statement

Wild-type (WT) AB zebrafish were obtained by the European Zebrafish Research Center (EZRC) (Germany). Zebrafish embryos were kept in petri dishes in zebrafish water (1.2 mM NaHCO_3_, instant ocean 0.1 g/L, 1.4 mM CaSO_4_, methylene blue 0.00002% w/v) at 28°C until 6 days post fertilization (dpf), then housed in ZebTEC semi-closed recirculation housing systems (Techniplast) at 28°C, pH 7.5 and conductivity 500 µS on a 14/10 light/dark cycle. Adult zebrafish were fed three times a day alternating dry food and brine shrimps. For the experiments, larvae and adult zebrafish were anesthetized using 0.016% w/v tricaine (3-amino benzoic acidethylester, Sigma Aldrich) in zebrafish water and sacrificed by tricaine overdose (0.03% w/v). All the experiments were performed in agreement with EU Directive 2010/63/EU. The experimental protocol was approved by Italian Ministry of Health (Approval Animal Protocol No.1191/2016-PR).

### 2.2 *In silico* analysis

Synteny maps of the chromosomic regions surrounding transmembrane protein 38A and transmembrane protein 38B (*TMEM38A/B*) genes in *D. rerio*, *H. sapiens* and *M. musculus* were generated using the human genes as reference by combining PhyloView and AlignView from Genomicus 93.01 (http://www.genomicus.biologie.ens.fr) with Ensembl Comparative Genomics data. Conserved domains between zebrafish, human and mouse proteins were identified using UniProt (https://www.uniprot.org).

### 2.3 *In situ* hybridization

Whole-mount *in situ* hybridization was carried out according to the standard protocol (21). An 841 bp amplicon was obtained by RT-PCR amplification of WT zebrafish *tmem38b* mRNA (ENSDART00000168983) using primers on exon 1 (5’-TCAATCTGAACGAGCTCGCATTT-3’, 20-42 nt) and on exon 10 (5’- AAGAAGCAGAAGCCAGCAAAAAG-3’, 839-861 nt) and subcloned in T7 pGEM-T-Easy vector (Promega). Plasmid DNA was linearized by enzymatic digestion with SacII (New England BioLabs) for the antisense RNA probe and with SpeI (Promega) for the sense probe. 48 and 72 hours post fertilization (hpf) embryos (n =10 for each group) were fixed in 4% (w/v) paraformaldehyde (PFA) o/n at 4°C, washed in PBS-T (PBS containing 0.1% Tween-20) and digested with 10 µg/mL proteinase K for a time depending on embryos’ developmental stage. Digoxigenin uridine-5′ triphosphate (DIG) labeled RNA sense and antisense probes targeting *tmem38b* gene were used after incubation at 64°C. Finally, images were acquired using a Leica M165 FC microscope connected to a Leica DFC425 C digital camera.

### 2.4 Genotyping

The WT, *tmem38b^-/-^* and *tmem38b^Δ120-7/120-7^* genomic DNA was extracted depending on the experiment type, from single embryos, pool of embryos or caudal fin biopsies from adult zebrafish. Tissues were digested by proteinase K (2.5 mg/mL, Sigma Aldrich) in lysis buffer (100 mM Tris HCl, pH 8.5, 5 mM EDTA, 0.2% (w/v) SDS, 200 mM NaCl) overnight (o/n) at 55°C, followed by isopropanol precipitation and resuspension in 20 mM Tris-HCl, 1 mM EDTA, pH 8.0. DNA was PCR amplified using the following primers: forward 5’-TTACTGTCCGCTGGATGTGG-3’ (11326-11345 nt) and reverse 5’–CAGAGCGTCGCTGTATTTGC-3’ (11448-11467 nt) as described in **Supplementary Methods**. The different amplicon sizes were discriminated on 12% and 10% v/v electrophoresis acrylamide gel in TBE buffer (0.1 M Tris HCl, 0.1 M H_3_BO_3_, 2 mM EDTA, pH 8.2), respectively.

### 2.5 RNA extraction and qPCR

RNA was extracted from RNA pools of 20 embryos at different stages of development (from 2-4 cells to 96 hours post fertilization, hpf) and from caudal fins of adult WT, *tmem38b^-/-^* and *tmem38b^Δ120-7/Δ120-7^* (WT n = 3, *tmem38b^-/-^* n = 3, *tmem38b^Δ120-7/Δ120-7^* n = 3) or from pools of bones, brains, muscles, swim bladders, hearts (WT n = 3 pools, *tmem38b^-/-^* n = 3 pools, *tmem38b^Δ120-7/Δ120-7^* n = 3 pools, each pool included samples from 2 fish) using Qiazol (Qiagen) and DNase digestion (Invitrogen) according to manufacturer’s instructions. All experiments were performed in triplicate, except when indicated in the figure legend. RNA quantity was determined by NanoDrop spectrophotometer and RNA quality by agarose gel electrophoresis. Reverse-transcription was performed using the High-Capacity cDNA Transcription kit (Applied Biosystems) according to manufacturer’s protocol in a final volume of 20 µL. qPCR was performed in triplicate in a 25 µL final volume using Taqman Universal PCR Master mix (Applied Biosystems) and commercial TaqMan probes for *tmem38b*, *tmem38a*, *rpl13a* and *β actin* (Dr03434781_m1, Dr03075180_m1, Dr03119260_g1 and Dr03432610_m1, Applied Biosystems). The relative expression of each gene was calculated using the ΔΔCt method. qPCR for *acp5a* (ENSDART00000004716.10), *bglap* (ENSDART00000100845.5), *ctsk* (ENSDART00000179680.1), *mpeg* (ENSDART00000077637.5), *opg* (ENSDART00000184909.1), *rankl* (ENSDART00000098355.5), *sp7* (ENSDART00000128793.3) and the housekeeping *dna15ta1* (22) was performed in 25 µL reaction mixtures with 12.5 µL SYBR Green Mastermix (Applied Biosystems). Primer sequences are available upon request. The QuantStudio 3 thermocycler and the QuantStudio Design and analysis software (Applied Biosystems) were used. Samples were run in triplicate.

### 2.6 Collagen analysis

Skin and bone were dissected from adult WT (n=2), *tmem38b^-/-^* (n=2) and *tmem38b^Δ120-7/Δ120-7^* (n=2) following sacrifice. The tissues were defatted for 6 h in 0.1 N NaOH at 4°C. Bone was decalcified for 48 h in 0.5 M EDTA pH 7.4 at 4°C. The pepsin-soluble collagen fraction (PSC) was obtained as described in (23). Briefly, tissues were digested with 0.1 mg/mL pepsin in 0.5 M acetic acid at 4°C for 48 h. The PSC was precipitated by 0.9 M NaCl in 0.5 M acetic acid o/n at 4°C and quantified using Sircol Soluble Collagen assay (Biocolor). Equal amount of collagen from each sample (2 µg) was loaded on 6% SDS-Urea-PAGE in non-reducing conditions. Gels were stained overnight with 0.08 M picric acid, 0.04% Coomassie Brilliant Blue R250 (Sigma Aldrich), destained in water and acquired with ImageQuant LAS 4000 (GE Healthcare) using the ImageQuant LAS 4000 1.2 software.

### 2.7 Morphometric analysis

Images of anesthetized post hatching stages (7, 14, 21 dpf and 1, 2, 4, 6 mpf) WT (n ≥ 13) and *tmem38b^-/-^* (n ≥ 8) larvae and adult zebrafish were acquired with a M165FC stereomicroscope (Leica) connected to DFC425C digital camera (Leica). For *tmem38b^Δ120-7/Δ120-7^*, images were taken at 21 dpf and 1 mpf (n ≥ 13). Measurements were performed as described (24) using the LAS v4.13 software (Leica). On lateral images, the Standard Length (SL) was measured as the distance from the snout to the caudal peduncle or, in pre-flexion larvae that do not have a caudal peduncle, to the posterior tip of the notochord. Vertebral length and height were evaluated at 21 dpf (WT n ≥22, *tmem38b^-/-^* n = 25, *tmem38b^Δ120-7/Δ120-7^* n = 19) and 1 mpf (WT n ≥18, *tmem38b^-/-^* n = 18, *tmem38b^Δ120-7/Δ120-7^* n = 18) in both mutants and also at 2 mpf in *tmem38b^-/-^* (WT n = 13, *tmem38b^-/-^* n = 14). For each vertebra, the mean of the length measured dorsally and ventrally, and the mean of the height measured anteriorly and posteriorly to the vertebral centrum were evaluated using the LAS v4.13 software (Leica). At 21 dpf, 10 vertebrae starting from the first ossified centrum were considered, while at 1 and 2 mpf the vertebral dimensions were evaluated by measuring from the second centrum articulated with the ribs. In addition, the level of inflation of the swim bladder was evaluated in larvae by counting the numbers of lobes on lateral images at 21 dpf (WT n ≥27, *tmem38b^-/-^* n = 26, *tmem38b^Δ120-7/Δ120-7^* n = 21).

### 2.8 Skeletal staining

Bone was stained with Alizarin Red as previously described (25). Both larval and adult WT (n ≥ 28), *tmem38b^-/-^* (n ≥ 17) and *tmem38b^Δ120-7/Δ120-7^* (n = 8) zebrafish were sacrificed and fixed overnight in PFA 4% w/v in PBS (Sigma Aldrich) with 0.9 mM CaCl_2_ and 0.49 mM MgCl_2_, pH 7.4 at 4°C. Bleaching to remove the pigmentation was performed with 3% v/v H_2_O_2_, 0.5% w/v KOH at RT followed by two washes in glycerol 25% v/v, 0.1% w/v KOH. Soft tissues were digested with 1 mg/mL trypsin dissolved in a 30% v/v solution of saturated B_4_Na_2_O_7_. After the staining in 0.01% w/v Alizarin Red S (Sigma Aldrich), 25% v/v glycerol, 100 mM Tris–HCl, pH 7.5 overnight at RT, fish were washed in increasing 0.1% series of glycerol/KOH and finally stored at 4°C in 100% glycerol, 0.1% KOH. Images were acquired using a Leica M165 FC microscope connected to a Leica DFC425 C digital camera. The mineralization of the notochord was evaluated describing the level of ossification as beginning, intermediate or complete ossification. The mineralization of Alizarin Red stained caudal fins was evaluated in the amputated samples and 5 days post amputation (dpa) (WT n ≥ 9, *tmem38b^-/-^* n ≥ 8, *tmem38b^Δ120-7/Δ120-7^* n ≥ 8) by measuring the real mineralized area (RMA) normalized to the ray width (RAY), according to literature (26). In addition, the length of segments per ray was measured on the amputated caudal fins (WT n = 6, *tmem38b^-/-^* n = 8, *tmem38b^Δ120-7/Δ120-7^* n = 3). Measurements were performed using the LAS v4.13 software (Leica).

### 2.9 Picro Sirius Red collagen staining

Amputated caudal fins of adult WT (n=3), *tmem38b^-/-^* (n=4) and *tmem38b^Δ120-7/Δ120-7^* (n=3) zebrafish were collected and fixed overnight in PFA 4% w/v in PBS. Caudal fins were stained 1 h in 0.1% w/v Sirius Red (Direct Red 80, Sigma Aldrich) in saturated aqueous solution of picric acid (Sigma Aldrich) (27). After staining, caudal fins were washed in 0.5% v/v acetic acid, and directly dehydrated three times in absolute ethanol. Samples were clarified with xylene and mounted with DPX (Sigma Aldrich). Slides were observed under polarized light with the DM2500 microscope (Leica) and acquired using the ICC50 W digital camera (Leica). Measurements were performed using Leica LAS v4.13 software on 20X images. First, the length of each actinotrichia per ray was measured by tracing a line from the most proximal red signal in the ray to the tip of the caudal fin. Then, all the actinotrichia whose length was measured were counted and the mean of the number of actinotrichia per ray was calculated.

### 2.10 Tartrate-resistant acid phosphatase staining

Tartrate-resistant Acid Phosphatase (TRAP) staining was performed (28). Caudal fins of adult WT (n = 7), *tmem38b^-/-^* (n = 9) and *tmem38b^Δ120-7/Δ120-7^* (n = 10) zebrafish were fixed in PFA 4% in PBS o/n at 4°C, washed in PBS containing 0.1% v/v Tween-20 and permeabilized in PBS containing 0.3% v/v Triton X-100 for 30 min. Fins were then equilibrated in TRAP buffer (0.1 M sodium acetate, 0.1 M acetic acid, 50 mM sodium tartrate) and colour reaction was performed in TRAP buffer containing 0.1 mg/ml Naphtol AS-MX phosphate (Sigma Aldrich) and 0.3 mg/ml Fast Red Violet LB (Sigma Aldrich). Fins were then bleached in 10% H_2_O_2_, 1% KOH o/n at RT to remove pigmentation and then stored in 70% glycerol at 4°C. Images were acquired using a Leica M165 FC microscope connected to a Leica DFC425 C digital camera. The number of TRAP+ cells in the regenerate was counted using the Cell Counter tool on the ImageJ software according to literature (29).

### 2.11 Transmission electron microscopy analysis

For transmission electron microscopy (TEM) analysis, 1 mpf WT (n= 3), *tmem38b^-/-^* (n=3) and *tmem38b^Δ120-7/Δ120-7^* (n=3) were fixed for 24 h at RT in 1.5% v/v PFA (Sigma Aldrich), 1.5% v/v glutaraldehyde (Sigma Aldrich), 0.1 M sodium cacodylate buffer (pH 7.4) and 0.001% w/v CaCl_2_. The samples were decalcified in 0.1 M EDTA for 14 days at 4°C. Samples were rinsed in 0.1 M sodium cacodylate buffer containing 10% sucrose and post fixed for 2 h using 1% w/v OsO_4_ in 0.1 M sodium cacodylate buffer at pH 7.4. Subsequently, zebrafish were infiltrated with low-viscosity epoxyembedding medium. Ultra-thin (70 nm) sections of the region of interest (vertebral endplate growth zone) were cut using a Reichert Ultracut E ultramicrotome (Reichert-Jung) with a diamond knife (Diatome Ltd.) and mounted on formvar-coated single slot copper grids. The sections were stained with uranyl acetate and lead citrate and viewed with a Jeol JEM-1010 (Jeol Ltd) TEM operating at 60 kV. Microphotographs were taken with a Veleta camera (Emsis, Germany) (30). TEM images were used to detect the endoplasmic reticulum cisternae enlargement. The area of ER cisternae in WT, *tmem38b^-/-^* and *tmem38b^Δ120-7/Δ120-7^* was measured using LAS v4.13 software (Leica).

### 2.12 4 phenylbutyrate treatment (4PBA)

WT, *tmem38b^-/-^* and *tmem38b^Δ120-7/Δ120-7^* embryos were manually dechorionated at 24 hpf. Fish were placed in 6 well plates (20 fish per well) in zebrafish water containing 0.003% 1-phenyl 2-thiourea (PTU) to prevent pigmentation and treated with 0.05 mM 4PBA from 1 dpf to 5 dpf. Half of the volume of water with or without 4PBA was replaced every day (23).

### 2.13 Whole-mount immunostaining

Whole mount immunostaining was performed as previously described (23). Briefly, *tmem38b^-/-^* (n=116) and WT (n=146) untreated and 4PBA treated *tmem38b^-/-^* (n=113); *tmem38b^Δ120-7/Δ120-7^* (n=19) and WT (n=43) untreated and 4PBA treated *tmem38b^Δ120-7/Δ120-7^* (n=34) were collected at 5 dpf, fixed overnight in 4% w/v PFA in PBS, washed in PBS and stored in methanol at -20°C. After tissue digestion with 0.1% w/v proteinase K in PBS at 25°C for 15 min and 2% w/v hyaluronidase in PBS at 25°C for 20 min samples were blocked in 5% w/v bovine serum albumin (BSA, Sigma Aldrich) in PBS-T for 2 h at RT. Hsp47b affinity purified antibody (1:1000 in 5% BSA/PBS-T), kindly provided by Prof. Raimund Wagener, University of Cologne, Germany, and anti-rabbit secondary antibody (1:200 in 1% BSA/PBS-T, Cell Signaling) were used. DAB substrate (Thermo Scientific) was finally added until appearance of the staining. Fish were incubated in increasing glycerol series and stored at 4°C in 100% glycerol. Images were acquired using a Leica M165 FC microscope connected to a Leica DFC425 C digital camera. Three operators blinded to the genotype and to the treatment of the fish independently evaluated the intensity of the signal as zero, low (+) or high (++). Following imaging, samples underwent stepwise ethanol dehydration and soft epon embedding according to an established protocol (31). Sagittal 4 μm sections were cut on a Microm HM360 microtome (Marshall Scientific), mounted with DPX and observed with an Axio Imager-Z1 microscope (Carl Zeiss).

### 2.14 Statistical analysis

All quantitative variables were expressed as mean with standard deviation (SD) or standard error of the means (SEM), as indicated in figure legend, while qualitative variables using percentage. To evaluate gene expression across genotype (WT, *tmem38b^-/-^* and *tmem38b^Δ120-7/Δ120-7^*) for each examined organ, non parametric analysis of variance (Kruskall Wallis) was applied, followed by non- parametric unpaired t test with multiple comparison correction. To separately describe the behaviour of WT with respect to each mutant in bone mineralization, swim bladder inflation and Hsp47 signal, chi-squared or Fisher’s exact test, if the assumption for chi squared was not respected, was performed. The Bonferroni’s correction for multiple tests was applied to adjust the p-value when the Hsp47 signal for untreated and treated mutants was compared to that of WT. Separately, by each time point a comparison between WT and *tmem38b^-/-^* standard length and vertebral measurements was evaluated using parametric or the equivalent non parametric unpaired t test, when the assumptions for parametric were violated. The same approach was applied to ray segment length, RMA/RAY width, number of TRAP positive cells and number and length of actinotrichia. A p<0.05 was considered significant apart from when multiple correction was used. Statistical analyses were performed using SigmaPlot and STATA15^®^.

## 3 Results

### 3.1 TRICs expression in zebrafish

**Figure 1:**
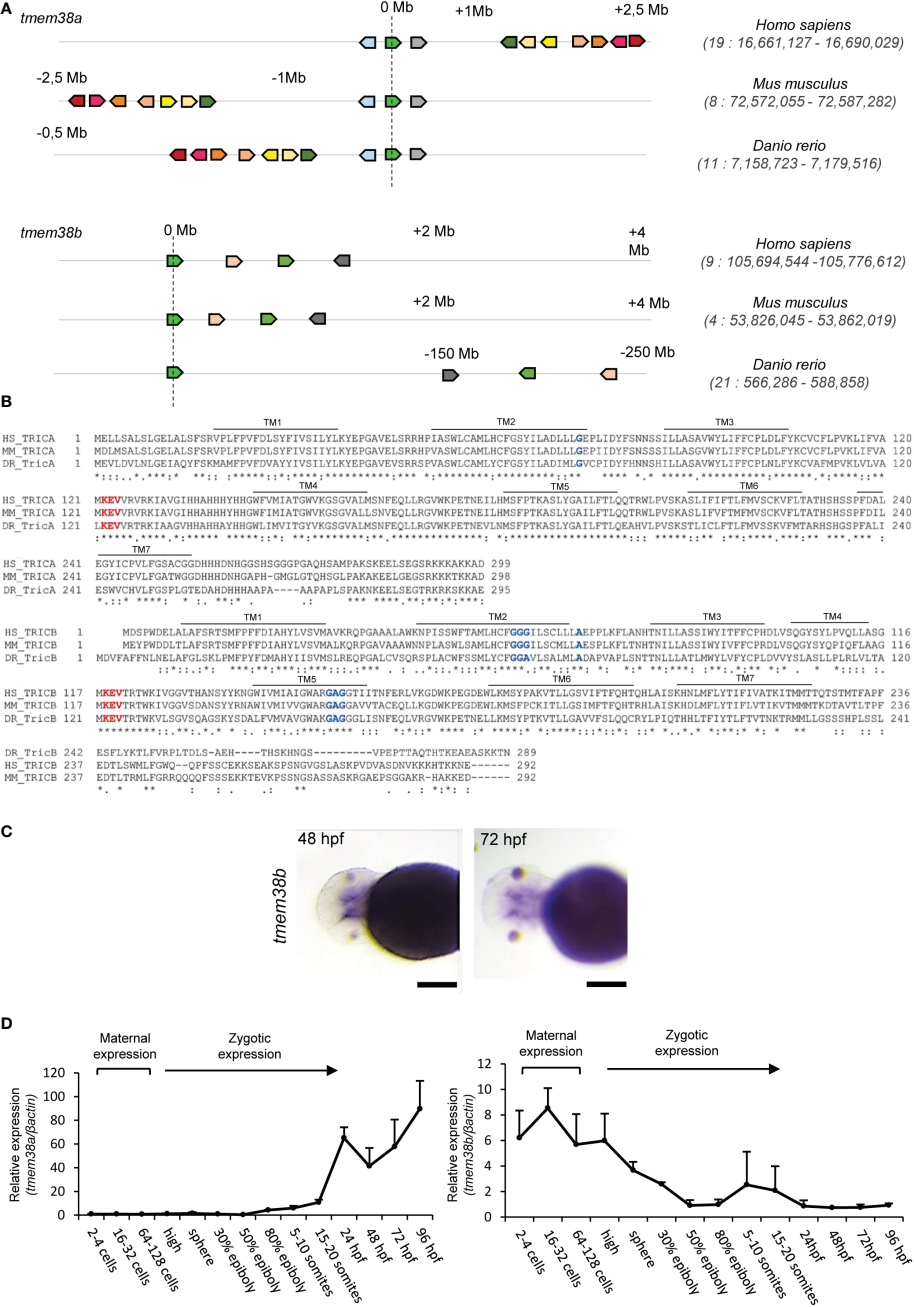
Analysis of *tmem38a/b* across species and of their spatio-temporal expression during zebrafish development. **(A)** The sinteny analysis performed on humans (*H. sapiens*), mice (*M. musculus*) and zebrafish (*D. rerio*) supported the existence of a common ancestral chromosomal origin for both t*mem38a* and *tmem38b*. The maps were obtained using the genome browser Genomicus. The human genes were used as roots. The position of the genes (Mb) relative to the investigated locus is based on Ensemble database and shown on top of the chromosome line. The exact chromosomal position of all the conserved genes is reported in the **Supplementary Tables S1**. The direction of the arrows indicates the gene orientation in respect to the reference gene. **(B)** Trimeric intracellular cation channel A (TRIC-A) and B (TRIC-B) domains are conserved among human, mouse and zebrafish. The 7 transmembrane domains (TM) are indicated. In red is shown the KEV pore channel domain, while in blue are indicated the two glycine reach regions in TM2 and TM5. **(C)** *In situ* hybridization analysis of 48 and 72 hpf WT embryos revealed the presence of *tmem38b* in the region of craniofacial cartilages. **(D)** Relative expression of *tmem38a* and *tmem38b* was evaluated by qPCR demonstrating a maternal expression only for *tmem38b*. The experiment was perfomed in duplicate. Scale bar: 500 µm. Human *TMEM38A* ENSG00000072954, murine *Tmem38a* ENSMUSG00000031791, zebrafish *tmem38a* ENSDARG00000024047; human *TMEM38B* ENSG00000095209, murine *Tmem38b* ENSMUSG00000028420, zebrafish *tmem38b* ENSDARG00000100549. Tric-a: human Q9H6F2, murine Q3PMT8, zebrafish Q6P2T0; Tric-b: human Q9NVV0, murine Q9DAV9, zebrafish Q7ZVP8.

A single copy of *tmem38a* and *tmem38b* encoding the trimeric intracellular cation channels (TRICs) Tric-a and Tric-b, respectively is present in the *D. rerio* genome. The zebrafish *tmem38a locus* is residing on chromosome 11 and similarly to the human and murine gene positioned on chromosome 19 and 8, respectively, consists of 6 exons. The zebrafish *tmem38b locus* is located on chromosome 21 and includes 10 exons, while the human and murine gene is located in chromosome 9 and 4, respectively and consist of 6 exons. The *in silico* analysis of the human, mouse and zebrafish genomic regions surrounding *tmem38a* and *tmem38b loci* supports their common ancestral origin (**Figure 1A** and **Supplementary Table 1**).

Zebrafish Tric-a consists of 295 amino acids and shows 70.6% and 68.1% identity with the human (299 aa) and the murine (298 aa) protein, respectively, whereas Tric-b (289 aa) shares only 45% and 48% identity with the human (291 aa) and the murine (292 aa) protein, respectively. Zebrafish Tric-a and Tric-b share 41.7% identity.

In all three species, homology data support that TRIC channels are symmetrical trimers of identical protomers crossing the endoplasmic reticulum membrane with 7 α helices and characterized by two inverted repeated regions and a C-terminal helix. Each protomer contains a lipid consensus binding sequence KEVXRXXK that likely confers to the channel the voltage-sensitivity and also includes the predicted pore-forming KEV, important to guarantee the K^+^ flux (32). Also, the two glycine rich regions in TM2 and TM5 are conserved as well as Gly74 in TRIC-A and Ala74 in TRIC-B that are recognized as luminal calcium binding site relevant for pore opening (32, 33) (**Figure 1B**).

**Figure 2:**
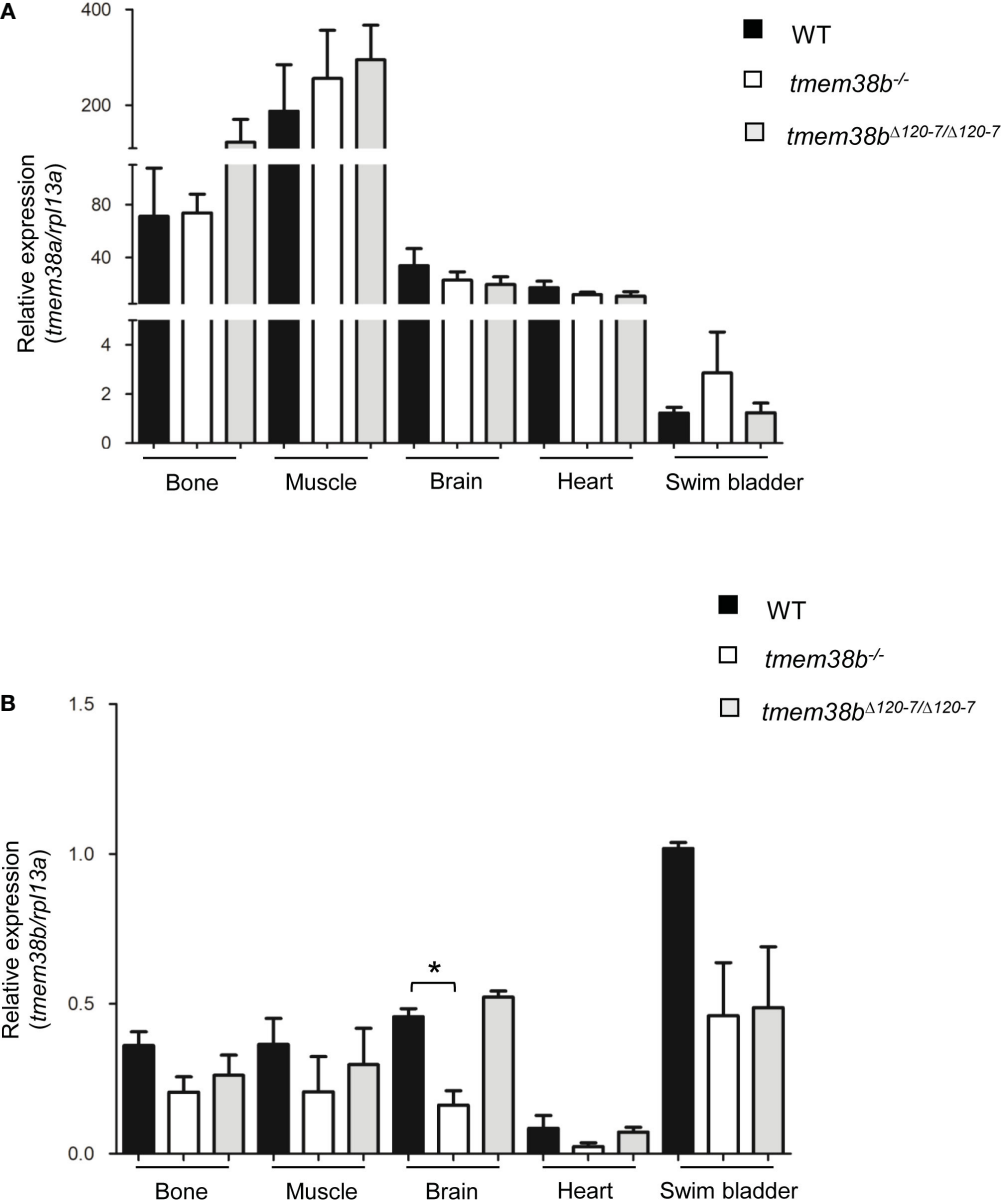
Expression analysis of *tmem38a* and *tmem38b* in excitable (muscle, brain, heart) and non-excitable tissues (bone, swim bladder) from adult WT and *tmem38b* mutants. **(A)** Relative expression of *tmem38a*. No difference in the transcript level was found between WT and *tmem38b* mutants by qPCR. **(B)** Relative expression of *tmem38b*. qPCR analysis revealed a reduced transcript level in almost all *tmem38b^-/-^* tissues respect to WT. Data are expressed as mean ± SEM. *p < 0.05 n = 3 RNA pools per group.

The expression of *tmem38b* at 48 and 72 hours post fertilization (hpf) in the region of cranio-facial cartilages was showed by whole mount *in situ* hybridization and supports also in teleosts the role of the channel in skeletal tissue starting from early developmental stage (**Figure 1C** and **Supplementary Figure 1A**). Temporal analysis of *tmem38a* and *tmem38b* expression in WT zebrafish at different embryonic developmental stages revealed a relevant increase of *tmem38a* expression from 24 hours post fertilization (hpf), while both a maternal and a zygotic expression were detected for *tmem38b* (**Figure 1D**). The two genes were expressed both in excitable (muscle, brain, heart) and in non-excitable (swim bladder, bone) adult tissues. As expected, the expression of *tmem38a* was particularly abundant in muscle, heart and brain, and surprisingly in bone as well, whereas the highest *tmem38b* expression was found in the swim bladder (**Figures 2A, B**).

### 3.2 *tmem38b* mutants generated by CRISPR/Cas9 gene editing

The skeletal phenotype described in osteogenesis imperfecta (OI) type XIV, and caused by *TMEM38B* loss-of-function mutations in mammals, demonstrated a relevant role of TRIC-B in bone homeostasis. Thus, to get further insight on *tmem38b* function in teleosts and to generate a zebrafish model for the human disease, CRISPR/Cas9 was used to target exon 7 (**Supplementary Figure 1B, C** and **Supplementary Materials**) which includes the consensus sequence KEVXRXXK important for the formation of the pore channel. Among the generated mutant F1 heterozygous zebrafish (**Supplementary Figure 1D**), two were selected and crossbred for further experiments. The one carrying a 7 nucleotides deletion (c.524_530delTGAAGGA) predicted to insert a premature stop codon at amino acid 122 of Tric-b was chosen to generate the F2 *tmem38b* knock out model (*tmem38b^-/-^*). The mutant carrying a 24 nucleotides *in frame* deletion (c.517_540del24nt) predicted to remove the p.Ala120_Thr127 oligopeptide was selected to generate the F2 *tmem38b^Δ120-7/Δ120-7^* lacking the highly conserved KEV motif (**Supplementary Figure 1D**). qPCR showed that *tmem38b* expression was significantly reduced in 24 hpf *tmem38b^-/-^* embryos compared to both WT (WT 1.19± 0.16, *tmem38b^-/-^* 0.40 ± 0.28, p=0.013) and *tmem38b^Δ120-7/Δ120-7^* (*tmem38b^-/-^* 0.40 ± 0.28, *tmem38b^Δ120-7/Δ120-7^* 2.11 ± 0.87; p= 0.031) while no difference in the expression of *tmem38a* was detected (WT 1.16± 0.14, *tmem38b^-/-^* 0.88 ± 0.12, *tmem38b^Δ120-7/Δ120-7^* 1.09 ± 0.17). In addition, the expression of *tmem38b* in various tissues revealed different levels of non-sense mediated mRNA decay (NMD) ranging from 41.2% in bone and muscle to 77.7% in heart for *tmem38b^-/-^* and from 16.6% in muscle and none in brain for *tmem38b^Δ120-7/Δ120-7^* (**Figure 2A, B**). Unfortunately, the lack of specific antibody against Tmem38b did not allow to evaluate the protein expression level.

### 3.3 Zebrafish juvenile skeleton is impaired in *tmem38b^-/-^*

**Figure 3:**
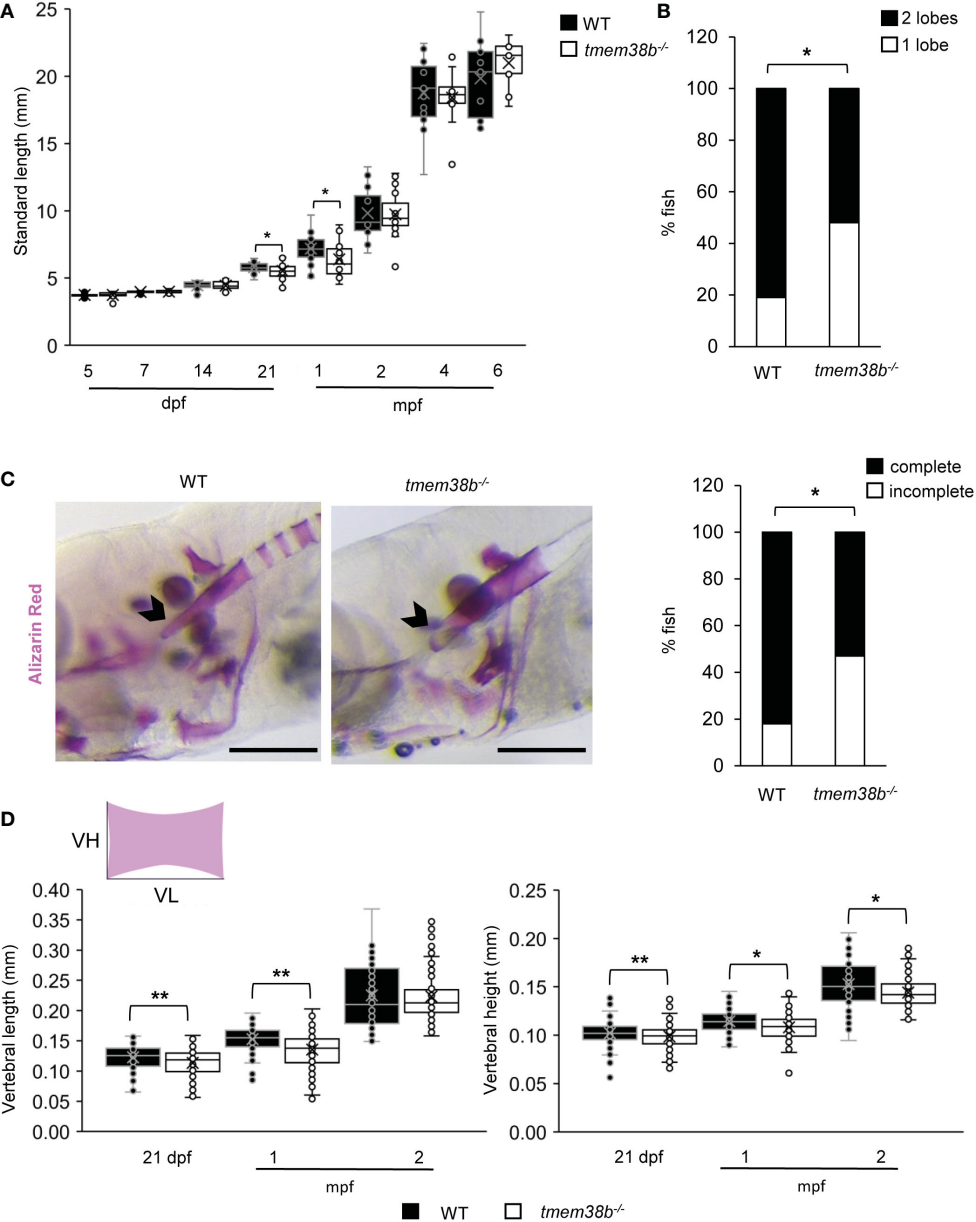
*tmem38b^-/-^* skeletal phenotype. **(A)** Growth curves representing WT and *tmem38b^-/-^* standard length measured at 5, 7, 14, 21 dpf, 1, 2, 4 and 6 mpf. At 21 dpf and 1 mpf *tmem38b^-/-^* were significantly shorter than WT. WT n ≥ 13, *tmem38b^-/-^* n ≥ 8. **(B)** At 21 dpf a significant delay in the inflation of the swim bladder was observed in *tmem38b^-/-^* (n = 27) with respect to WT (n = 28). **(C)** Representative lateral view (left panels) of 14 dpf alizarin red stained WT and *tmem38b^-/-^* fish. The notochord is indicated by the arrowhead. On the right the graph represents the level of the tip of the notochord mineralization, a delayed mineralization was evident in *tmem38b^-/-^* (*tmem38b^-/-^* n = 17) respect to WT (WT n = 28) Scale bar: 500 µm. **(D)** Vertebral length and vertebral height measurement in WT and *tmem38b^-/-^* zebrafish. Vertebral length (VL) was reduced in *tmem38b^-/-^* compared to WT at 21 dpf and 1 mpf. Vertebral height (VH) was reduced in *tmem38b^-/-^* compared to WT at 21 dpf, 1 and 2 mpf. 21 dpf: WT n= 29; *tmem38b^-/-^* n= 25; 1 mpf: WT n= 24; *tmem38b^-/-^* n= 18; 2 mpf: WT n= 13; *tmem38b^-/-^* n= 14. *,p < 0.05, **p ≤ 0.01.

Both *tmem38b* mutants were viable, reached adulthood and were fertile. Nevertheless, an increased lethality was observed in *tmem38b^-/-^* from 7 days post fertilization (dpf) compared to both WT and *tmem38b^+/-^*. Indeed, a significant difference from the expected 1:2:1 Mendelian ratio was observed at 2 and 3 weeks of age (**Supplementary Table 2**). The growth curve followed from 5 dpf to 6 months post fertilization (mpf) revealed a significant reduction in the standard length (SL) at 21 dpf and 1 mpf in *tmem38b^-/-^* compared to WT (**Figure 3A**) and a delay in the inflation of swim bladder lobes was detectable in mutant fish at 21dpf, suggesting a developmental delay rescued at older age (**Figure 3B**). No difference in SL and in the swim bladder inflation was evident in *tmem38b^Δ120-7/Δ120-7^* (**Supplementary Figures 2A, B**).

Whole mount Alcian Blue and Alizarin Red staining of 5 and 7 dpf larvae, respectively, did not reveal abnormality in cartilage or skeletal development in *tmem38b^-/-^* compared to WT (**Supplementary Figures 3A-D**). A reduced level of mineralization in the tip of notochord sheath was observed in *tmem38b^-/-^* compared to WT at 14 dpf (**Figure 3C**). An impaired mineralization of vertebra centra at 21 dpf and 1 mpf, as demonstrated by reduced vertebral length and vertebral height, the latter being smaller also at 2 mpf, was also detected (**Figure 3D**). Double staining with alizarin red and calcein did not show any difference in bone formation rate between WT and *tmem38b^-/-^* from 10 dpf to 1mpf (**Supplementary Figure 4**). The mineralization in mutant fish was partially rescued at 4 mpf, when only vertebral height was still reduced (**Supplementary Figure 2C**).

The vertebral size was unchanged in the *tmem38b^Δ120-7/Δ120-7^* at the analyzed time points, with the exception of a reduced vertebral length at 21 dpf (**Supplementary Figure 2D**).

MicroCT on both adult (9 mpf) fish models did not reveal any alteration in bone geometrical parameters compared to WT (**Supplementary Figure 5** and data not shown). Also, nanoindentation analysis performed on vertebral bone cortex of 2 mpf mutants and control fish showed no differences either in hardness (*tmem38b^-/-^* 0.598 ± 0.050 GPa; *tmem38b^Δ120-7/Δ120-7^* 0.628 ± 0.068 GPa; WT 0.642 ± 0.052 GPa) or in elastic modulus (*tmem38b^-/-^* 13.704 ± 0.665 GPa; *tmem38b^Δ120-7/Δ120-7^* 13.895 ± 1.029 GPa; WT 14.391 ± 0.795 GPa).

Taken together, the skeletal characterization revealed a mild effect of Tric-b absence in zebrafish endoskeleton limited to late larval-juvenile developmental stage when a rapid bone growth is required. The lack of bone phenotype in *tmem38b^Δ120-7/Δ120-7^* supports either a possible residual activity of the *in frame* mutant Tric-b channel.

### 3.4 Collagen type I is retained in the endoplasmic reticulum of *tmem38b* mutants

**Figure 4:**
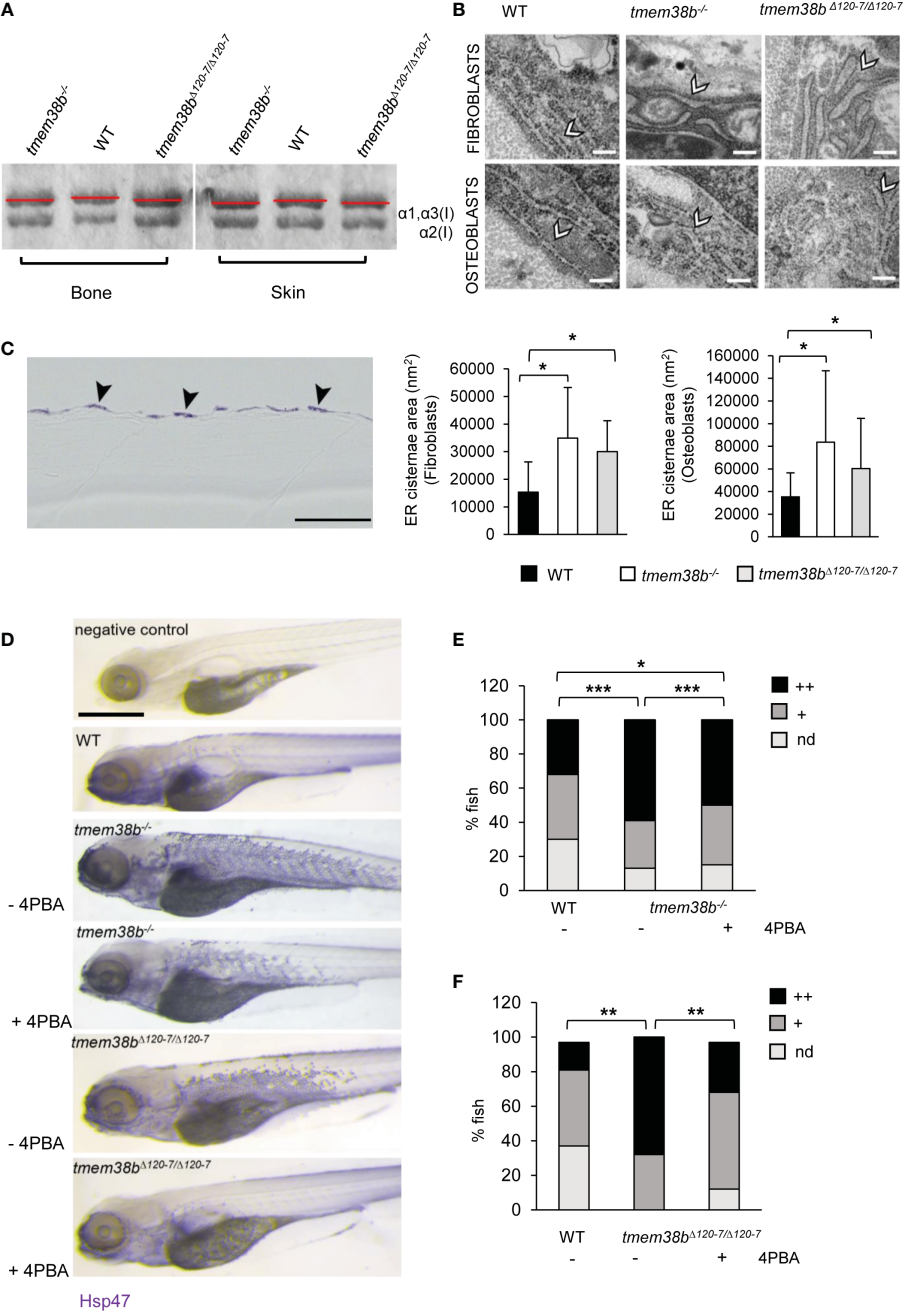
Collagen type I and ultrastructural analysis. **(A)** Representative Coomassie stained SDS-Urea-PAGE of collagen type I extracted from WT (n = 2) and mutants (*tmem38b^-/-^* n = 2; *tmem38b^Δ120-7/Δ120-7^* n = 2) bone and skin. Mutants’ collagen α bands presented a slight faster migration compared to WT as highlighted by red lines. **(B)** Transmission electron microscopy images of 1 mpf WT (n = 3), *tmem38b^-/-^* (n = 3), and *tmem38b^Δ120-7/Δ120-7^* (n = 3) fibroblasts and osteoblasts at the vertebral endplate. ER cisternae enlargement was evident in mutants (arrowheads). Magnification 80000x. Scale bar: 200 nm. The ER cisternae area quantitation is shown in the graphs (bottom). **(C)** Immunohistochemistry of 5 dpf WT and *tmem38b^-/-^* with Hsp47b antibody. High magnification details of the skin of WT and *tmem38b^-/-^* following immunostaining with Hsp47b antibody revaled that Hsp47 signal was located in the fibroblasts of the skin (arrowheads). (**D)** Representative images of 5 dpf fish after whole mount immunohistochemistry with Hsp47b antibody, before and after 4PBA administration. Scale bar: 500 μm. **(E)** Analysis of Hsp47b expression by whole mount immunohistochemistry in WT and in *tmem38b^-/-^* before and after 4PBA administration. A significant increase of Hsp47 signal was detected in *tmem38^-/-^* compared to WT. 4PBA treatment significantly reduced the Hsp47b signal in *tmem38b^-/-^* compared to untreated mutant fish without reaching WT value (WT n = 146 and *tmem38b^-/-^* n = 116 untreated and 4PBA treated *tmem38b^-/-^* n = 113). **(F)** Analysis of Hsp47b expression by whole mount immunohistochemistry in WT and in *tmem38b^Δ120-7/Δ120-7^* before and after 4PBA administration. A significant increase of Hsp47 signal was present in *tmem38b^Δ120-7/Δ120-7^* compared to WT. 4PBA treatment significantly reduced the Hsp47b signal in *tmem38b^Δ120-7/Δ120-7^* compared to untreated mutant fish reaching WT value (WT n = 43 and *tmem38b^Δ120-7/Δ120-7^* n = 19 untreated and 4PBA treated *tmem38b^Δ120-7/Δ120-7^* n = 34). Zero (nd), low (+) and high (++) indicate the intensity of the signal. *p < 0.05, **p ≤ 0.01, ***p ≤ 0.001.

Collagen type I extracted from WT and both mutants’ skin and bone showed a slight faster electrophoretic migration (**Figure 4A** and **Supplementary Figure 6A**) supporting the presence of collagen under-modification, as reported in human OI type XIV cells (17). Electron microscopy analysis performed in the vertebral region revealed enlarged ER cisternae size in both fibroblasts and osteoblasts (**Figure 4B**) of *tmem38b* mutants compared to WT.

The expression of Hsp47b, the collagen specific chaperone known to assist procollagen assembly in the ER and its trafficking into the Golgi and whose expression is coupled with collagen synthesis (34), was evaluated by whole mount immunostaining on 5 dpf larvae.

Histological sections revealed that Hsp47b signal is intracellular and expressed in the outer epidermal layer (**Figure 4C**).

A stronger and significantly higher Hsp47b signal in mutants compared to WT was detected (**Figures 4D–F**).

Interestingly, the administration of 4 phenyl butyrate (4PBA), a chemical chaperone known to release ER stress in presence of collagen accumulation in dominant (23, 35) and some OI recessive forms (36), reduced Hsp47 signal in both mutants compared to controls supporting its positive role in restoring cell homeostasis also in presence of *tmem38b* mutations (**Figures 4D-F**).

### 3.5 Caudal fin rays and their regeneration are impaired in *tmem38b^-/-^*

**Figure 5:**
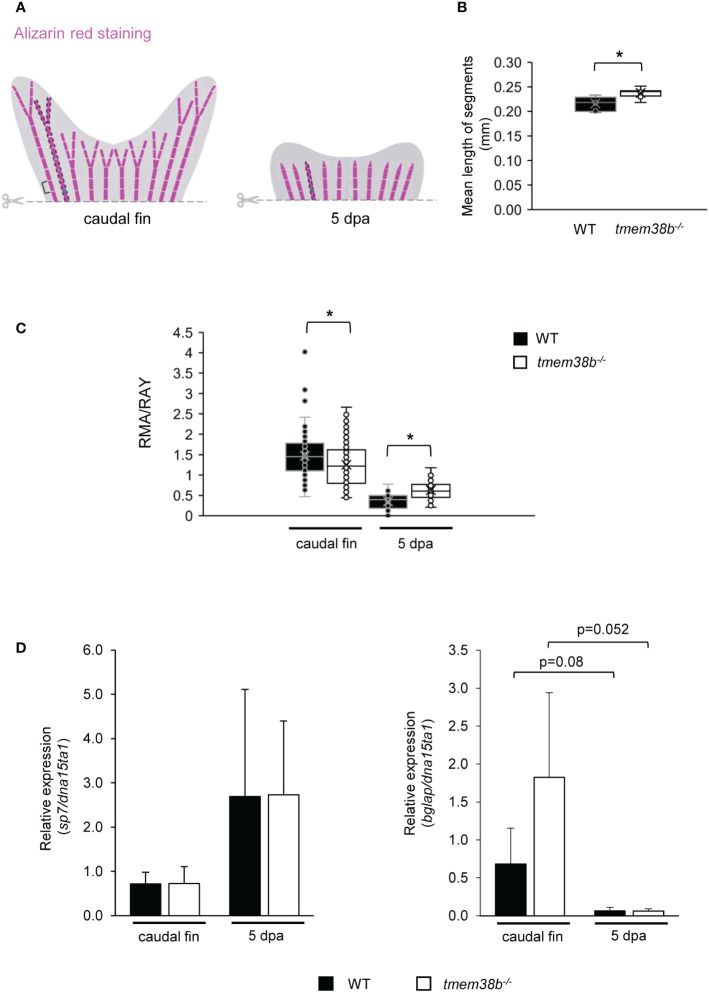
Morphometric and bone mineralization analyses during caudal fin regeneration in WT and *tmem38b^-/-^* mutants. **(A)** Schematic representation of morphometric measurements. Brackets indicate the length of fin ray segment, dashed areas represent the real mineralized areas (RMA) and the green lines indicate the ray widths (RAY). **(B)** The mean length of caudal fin ray segments was evaluated. Segments were significantly longer in *tmem38b^-/-^* compared to WT. **(C)** The ratio between the real mineralized area (RMA) and the mean ray width (RAY) was measured on alizarin red stained caudal fins to assess mineralization level. *tmem38b^-/-^* caudal fin was less mineralized respect to the WT. After 5 dpa, the regenerated mineralized area was larger in *tmem38b^-/-^* respect to the WT (WT n ≥ 9, *tmem38b^-/-^* n ≥ 8) *p < 0.05. **(D)** Relative expression of the early (*sp7*) and late (*bglap*) osteoblastic markers in WT and *tmem38b^-/-^* caudal fins in amputated and 5 dpa (WT n = 3, *tmem38b^-/-^* n = 3) samples. No significant difference was detected in *sp7* and *bglap* expression between WT and *tmem38b^-/-^* at both time points, even if a trend towards higher value was evident for *bglap* in mutant caudal fins with respect to controls. Data are shown as mean ± SEM.

To evaluate the role of Tric-b during bone formation, caudal fin regeneration experiments were carried out. First, zebrafish amputated caudal fins were analyzed following Alizarin Red staining and a significantly increased segment length was detected in *tmem38b^-/-^* compared to controls (**Figures 5A, B**). In amputated and regrown samples stained with alizarin red the real mineralization area (RMA) was evaluated for all fin rays and normalized to ray width (RAY) (26). In *tmem38b^-/-^* a reduced RMA compared to WT was evident in the amputated samples, whereas at 5 days post amputation (dpa) it was significantly increased, suggesting a mineralization boost during early bone formation (**Figures 5A, C**).

qPCR analysis on WT and *tmem38b^-/-^* pools of caudal fins showed no difference in the expression of the early osteogenic marker *sp7* (*osterix*) at both time points. The increased expression trend of the late marker *osteocalcin* (*bglap*) in the mutant amputated fins compared to WT normalized in 5 dpa regenerates (**Figure 5D**).

No difference in bone mineralization during caudal fin regeneration as well as in the expression of specific markers of bone differentiation was detected in *tmem38b^Δ120-7/Δ120-7^* compared to WT (**Supplementary Figures 6B–D**).

### 3.6 Analysis of bone resorption during caudal fin regeneration in *tmem38b* mutants

**Figure 6:**
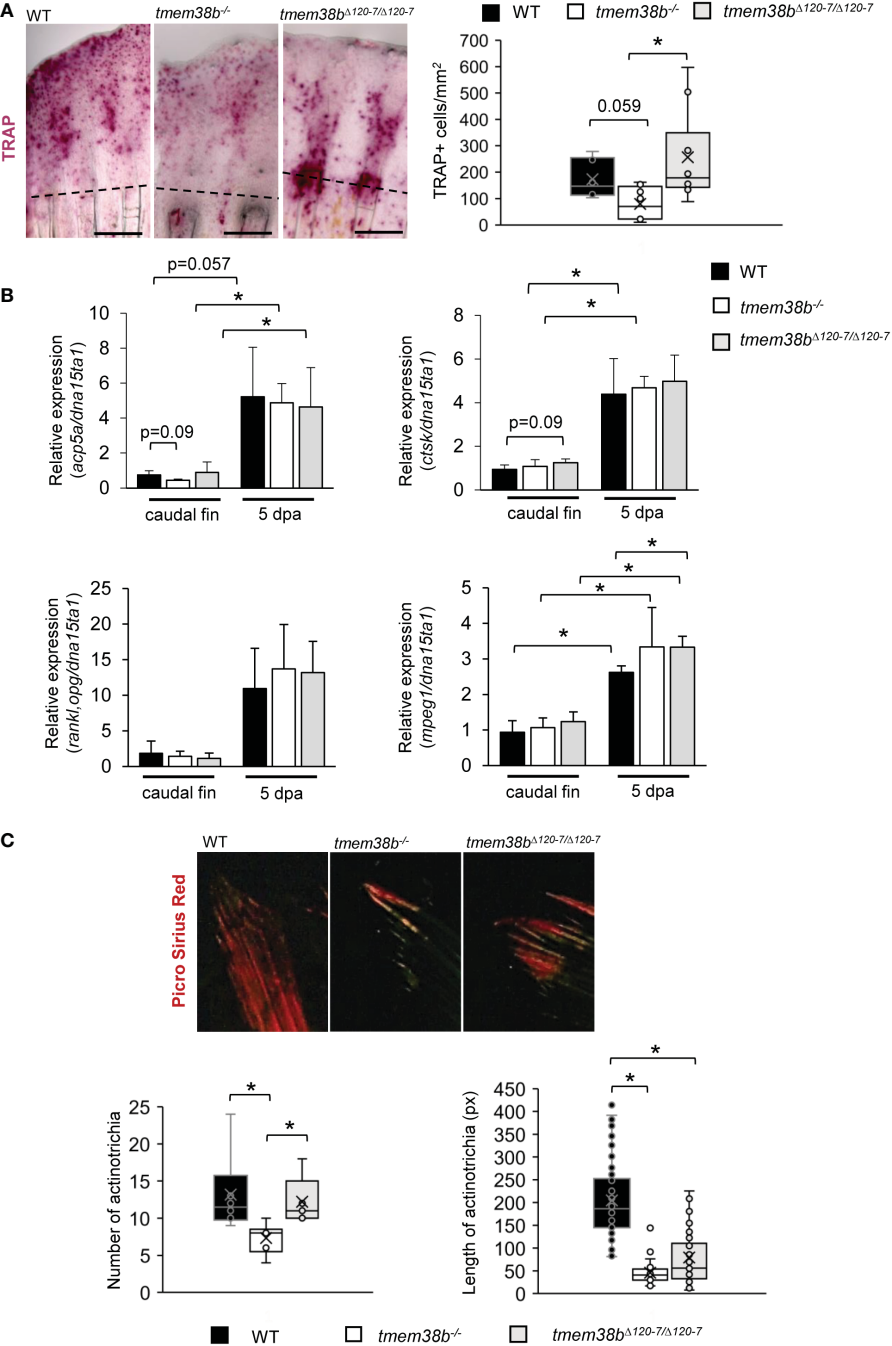
Osteoclast analysis during caudal fin regeneration in *tmem38b* mutants. **(A)** Representative images of TRAP staining of caudal fins of WT and *tmem38b* mutants at 5 dpa (WT n = 7, *tmem38b^-/-^* n = 9, *tmem38b^Δ120-7/Δ120-7^* n = 10). In WT, osteoclasts were present in the regenerate. Almost no TRAP activity could be detected in *tmem38b^-/-^*, while in *tmem38b^Δ120-7/Δ120-7^* it seemed to be mostly localized along the regenerating fin rays. TRAP+ cells were significantly reduced in *tmem38b^-/-^* respect to the other two groups. **(B)** Relative expression of bone resorption-related markers *acp5a* and *ctsk*, of the macrophage marker *mpeg1* and *rankl*/*opg* ratio in amputated and 5 dpa caudal fin (WT n = 3, *tmem38b^-/-^* n = 3, *tmem38b^Δ120-7/Δ120-7^* n = 3). All markers were increased after amputation. *mpeg1* was significantly overexpressed in *tmem38b^Δ120-7/Δ120-7^* compared to WT at 5 dpa.*: p < 0.05. **(C)** Representative images of picro sirius red staining of actinotrichia in caudal fins of WT and *tmem38b* mutants (WT n = 3, *tmem38b^-/-^* n = 3, *tmem38b^Δ120-7/Δ120-7^* n = 3). *tmem38b^-/-^* revealed a reduced number of actinotrichia respect to WT and *tmem38b^Δ120-7/Δ120-7^*, while the length of actinotrichia was reduced in both mutants compared to WT. *p < 0.05.

Osteoblast and osteoclast activity in bone is tightly coupled and OI mutations frequently lead to an imbalance between bone formation and bone resorption (37). Indeed, in OI type XIV individuals a reduced number of osteoclasts associated to bone resorption was observed (16). To investigate bone resorption in *tmem38b* mutants both histomorphometric and molecular analyses were performed in regenerated caudal fins. Tartrate resistant acid phosphatase (TRAP) staining revealed a significant reduction in the number of TRAP+ cells in the 5 dpa *tmem38b^-/-^* regenerated caudal fin compared to WT. Interestingly, a strong TRAP staining signal was detected at the amputation plane and in the region corresponding to the new forming rays in *tmem38b^Δ120-7/Δ120-7^*. On the contrary of WT and *tmem38b^-/-^*, no signal was evident in the distal tip of the fin (**Figure 6A**). Nevertheless, *tmem38b^Δ120-7/Δ120-7^* and WT had similar number of TRAP+ cells.

No difference in the expression of the main osteoclast markers, *acp5a* (encoding for Trap), *ctsk* and in the ratio *rankl/opg* was detected in both mutants compared to WT (**Figure 6B**). An increased expression of the macrophage marker *mpeg1* was detected in *tmem38b^Δ120-7/Δ120-7^* respect to WT at 5 dpa, suggesting the presence of high number of osteoclast precursors (**Figure 6B**).

### 3.7 Actinotrichia formation is impaired in *tmem38b* mutants

Actinotrichia, spear-like structures made of collagen I, collagen II and actinodins 1 and 2 proteins located at the tip of each caudal fin ray, play a morphogenetic role in fin formation, representing a guide for osteoblasts distal migration although synthesized by different cell types, probably basal keratinocytes (27, 38, 39). Upon Picro Sirius Red staining of caudal fins WT and *tmem38b* actinotrichia number and length were evaluated. Polarized light microscopy demonstrated a reduced length of actinotrichia in both mutants compared to WT and limited to *tmem38b^-/-^* also a significant reduction in their number (**Figure 6C**).

## 4 Discussion

The present study demonstrates, for the first time to the best of our knowledge, the relevance of Tric-b in zebrafish bone during development and bone cells differentiation, providing further insight on the relevance of this channel for the skeleton and supporting a shared role between teleosts and mammals. Both *in silico* and *in vivo* approaches were applied.

### 4.1 Trics are conserved in zebrafish

The retention of trimeric intracellular cation-specific channels TRIC-A and TRIC-B in archaea, bacteria and eukaryotes underlines their relevance in the animal kingdom, and their high level of homology throughout species supports a common phylogenetic origin (40). Indeed, the synteny analysis performed around zebrafish *tmem38a* and *tmem38b loci* demonstrated the presence of conserved synteny blocks shared with *M. musculus* and *H. sapiens*. The identity is higher for TRIC-A than for TRIC-B, nevertheless, in each protomer all the critical amino acids necessary for pore formation and for protomer association into the homotrimeric channels are present (33). In particular, zebrafish Tric sequence contains the glycine rich domains, responsible for the intramembrane kinks interacting with diacylglycerol in the transmembrane helices (TM) 2 and 5 and the voltage sensing domain in TM4, where also the K129 gating residue, corresponding to K125 in *H. sapiens* and *M. musculus*, is conserved.

TRICs function in prokaryotes is still unclear, whereas in eukaryotes their localization as integral membrane protein of the sarcoplasmic/endoplasmic reticulum (SR/ER) and their specificity for monovalent cations, in particular potassium, support their function as counter ion channels contributing to maintain the SR/ER membrane electro-neutrality following calcium release. Indeed, *in vitro* and *in vivo* studies in human and murine cells, confirmed an impaired SR/ER calcium release in their absence (6, 8, 17, 41).

Of note, both in invertebrates and in vertebrates, after sperm fertilization Ca^2+^ waves regulate the polarization of cytoplasmic domains in oocytes and drive early embryo patterning and subsequent development (42–46). This early event depends largely on maternal gene products (47). The calcium responsible for egg activation originates from intracellular storage, mainly the endoplasmic reticulum, through the activity of phospholipase C (PLC) and inositol-tris-phosphate (IP3) (45, 48), the specific ligand allowing the opening of the inositol 3 phosphate receptor (IP3R) ER calcium channel. Between the TRIC channel subtypes, TRIC-A and TRIC-B, is the latter, ubiquitously distributed, that is coupled with IP3R, whereas TRIC-A, most abundantly present in excitable cells, is coupled with ryanodine receptors (RyRs) (2). We first demonstrated that in zebrafish both channels are expressed at early developmental stages, but only *tmem38b* had a maternal expression, supporting its association with IP3R also in teleosts and its relevance during the first stage of embryo formation. On the contrary, *tmem38a* RNA was detectable only starting from 24 hpf at the appearance of somites, thus confirming the previous study on single-cell gene mapping expression (49), and in adult was present in all excitable and not-excitable analyzed tissue. As in mammals, the highest level of *tmem38a* mRNA in zebrafish is in skeletal muscle, but it is also expressed in bone (1, 50).

### 4.2 Tric-b is necessary in zebrafish for proper bone formation during fast growth developmental stages

In 2014, the identification of *TMEM38B* loss-of-function mutations in individuals affected by the recessive form of osteogenesis imperfecta (OI) type XIV demonstrated an unexpected and relevant role for the ER ion channel TRIC-B in mammalian bone homeostasis (8). Similarly, *Tmem38b * knock out mouse showed bone defects, even if mutant mice are perinatally lethal due to impairment in surfactant production, making difficult to characterize the murine skeletal outcome and its molecular basis (8).

Taking into account the high TRIC-B homology in teleosts and mammals and the suitability of zebrafish as a model for dominant and recessive OI forms (23, 25, 51), we targeted zebrafish *tmem38b* by CRISPR/Cas9 both to understand the role of Tric-b in *D. rerio* and to generate the first teleost model for the human disease. Two mutants were created and deeply characterized, the first one carrying a frameshift mutation (*tmem38b^-/-^*) associated to the insertion of pre-termination codon and the second carrying a 24 bp *in frame* deletion causing the removal of a consensus sequence relevant for channel activity in the voltage-sensing TM4 domain (*tmem38b^Δ120-7/Δ120-7^*). The highly conserved K122 and R126 amino acids are also lost in this model. On the other hand, the K129 residue with a relevant role in gating Tric channels is kept in *tmem38b^Δ120-7/Δ120-7^* leaving open the possibility of a residual activity for the translated protein.

The activation of nonsense mediated mRNA decay (NMD) of mutant mRNA, a process generally associated to the insertion of a premature stop codon, was proved in *tmem38b^-/-^*, and absent in *tmem38b^Δ120-7/Δ120-7^*. Interestingly, *tmem38b^-/-^* NMD was tissue dependent with a mean value of 69% in excitable tissues (muscle, brain, heart) and of 75% in non-excitable tissues (bone and swim-bladder). Tissue specific NMD was previously described in murine models and could indeed be relevant for understanding the genotype-phenotype relationship and the severity in heritable diseases (52, 53). Of note, NMD in bone was below 50% supporting the possibility of a certain level of translation of the truncated protein.

Unfortunately, despite the multiple attempts, in none of the models the protein expression could be evaluated due to the lack of specific antibody.

No compensatory effect of the *tmem38a* transcript was observed during early stage of development (24 hpf) in mutants compared to WT. Moreover, the increase of *tmem38a e*xpression (≥ 1.8 fold) detected in bone and muscle of *tmem38b^Δ120-7/Δ120-7^* during adulthood did not reach significant value.

Fish standard length and vertebrae size evaluated at several developmental stages were reduced in *tmem38b^-/-^* only at 21 dpf and 1 mpf. These time points overlap with the larval to juvenile transition that is associated to quick and significant body growth (24), likely demanding Tric-b activity and thus supporting a relevant role of *tmem38b* for early bone development rather than for adult skeletal homeostasis. Indeed, active skeletal growth implies a strong osteoblast activity and thus collagen synthesis and secretion, which indeed have been demonstrated to be altered in *TMEM38B* knock out human and mice cells (8, 17). Nevertheless, no difference in BFR was detectable between *tmem38b^-/-^* and WT from 10 dpf to 1mpf. *Tmem38b^-/-^* body length and vertebral size reach and maintain WT values in adult, in agreement with the reduced fracture frequency and bone properties amelioration after puberty described in OI patients (9, 54, 55). The skeletal phenotype in *tmem38b^Δ120-7/Δ120-7^*, limited to reduced vertebral length at 21 dpf, supported a translation and likely a partial activity of Tric-b in the model and let us to hypothesize that a full active channel is necessary for proper bone formation.

*Tmem38b^-/-^* and *tmem38b^Δ120-7/Δ120-7^* geometrical and material bone properties revealed by microCT and nanoindentation analysis at vertebral sites were in the normal range, but it should be taken into account that the very small zebrafish size at younger stages did not allow us, due to technical limitations, to evaluate bone properties at 21 dpf and 1 mpf, when differences in length and vertebral size were detectable.

### 4.3 Tric-b is required for proper swim bladder inflation

Fish swim bladder originates from the foregut endoderm, representing an evolutionary similarity with human lungs and, in addition, the presence of collagen type I was described at least in seabass (*Lates calcarifer*) (56). Mutations in *TMEM38B* are associated with pulmonary dysfunction both in OI type XIV individuals and in the *Tric-B* knock out mouse model, which dies immediately after birth due to respiratory failure (8, 16). Interestingly, *tmem38b^-/-^* revealed a delay in the inflation of the second lobe of the swim bladder in 21 dpf larvae compared to WT supporting Tric-b requirement for proper organ development, even if its direct role as potassium channel or indirect activity in collagen I synthesis are still unclear. A delayed swim bladder inflation was already described in the recessive OI type VII and VIII zebrafish models, carrying loss-of-function mutations in *crtap* and *p3h1*, respectively (25) and the complete lack of swim bladder inflation was reported in *col1a1a^-/-^* (51) proving that a normal amount and/or structure of collagen I in its extracellular matrix is necessary for proper swim bladder inflation. The lack of swim bladder defect in *tmem38b^Δ120-7/Δ120-7^* further supports in the model the presence of a translated and partial active Tric-b, pointing to its requirement for fish development.

### 4.4 Tric-b plays a role in dermal exoskeletal appendices

Histomorphometric analysis of caudal fin rays in adult mutants revealed a high variability in caudal fin ray segment length with an overall significantly higher mean in *tmem38b^-/-^* compared to control and *tmem38b^Δ120-7/Δ120-7^*. Fin ray segment length was associated to the conductance activity of Kcnk5b, a plasma membrane potassium channel belonging to the two pore family of channels. Gain-of-function mutations in *kcnk5b* are responsible for the *another longfin* (*alf*) zebrafish phenotype characterized by increased fin ray segment length (57). The hyperpolarization of the membrane consequent to these mutations represents a proof of the relevance of bioelectric signals not only in excitable tissues, but also for development and physiology in the non-excitable ones. Similarly to *tmem38b^-/-^*, the *kcnk5b* mutants are viable and fertile. Interestingly, the activity of Kcnk5b is modulated by calcineurin that acts as channel inhibitor by binding to the cytosolic C-terminal end of the channel (58). Indeed, treatment of WT zebrafish with the calcineurin inhibitor FK506 well reproduces the *alf* phenotype (59). Calcineurin is a calcium dependent protein, whose activity is strongly dependent from intracellular calcium concentration (60). In mammals, the absence of TRIC-B, acting as counter ion for calcium flux from the ER, has been reported to decrease the calcium cytosolic concentration (17), thus the longer *tmem38b^-/-^* fin ray segments may indeed be the natural consequence of that, supporting a shared role of Tric-b between mammals and teleosts.

In *alf* mutants the longer segments are associated to an overall increase of caudal fin length whereas in *tmem38b^-/-^* mutant caudal fin size is within normal values (data not shown). Nevertheless, in *tmem38b* mutants a reduced length of actinotrichia is reported, and in *tmem38b^-/-^* also the number is reduced indicating that either reduction or lack of Tric-b impair their formation. The actinotrichia are spear-like structures containing collagen I located at the tip of each caudal fin ray (27). Fins grow through sequential addition of lepidotrichial segments at their distal tip *via* migration of mesenchymal cells along the actinotrichia, clusters of collagenous fibers that emerge from the tip of each lepidotrichium (61, 62). Thus, reduced length/number of actinotrichia could indeed negatively affect tail growth counteracting the longer segments.

### 4.5 Tric-b plays a role in osteoblasts differentiation

To address the effect of the absence of *tmem38b* on bone cells differentiation, caudal fin regeneration, a well-organized process that partially recapitulates the events occurring during bone development, was employed. The similar expression of *sp7* both in the amputated samples and 5 dpa regenerates in mutants and controls suggested that the reduced ray mineralization detected in *tmem38b^-/-^* caudal fin compared to WT was not a consequence of impairments on osteoblast de- and early re-differentiation. The overexpression of *bglap*, previously described in OI type XIV cultured primary osteoblasts as well as in the *TMEM38B* knock out human foetal osteoblasts (hFOB), may be related to the inhibition of hydroxyapatite crystal growth (16, 63) and could indeed explain the reduced alizarin red staining of caudal fin in the amputates samples. Less clear is the increased mineralization during early regeneration phase, suggesting an accumulation of minerals during bone modelling that could be due either to an impaired cellular function or to an increased inter-fibrillar spacing that undergoes remodelling during growth.

The effect of *tmem38b* targeting on osteoclasts (OCs) was also addressed using the amputation assay since osteoclasts are involved in zebrafish fin ray healing at the amputation site as well as at newly regenerated rays (28).

In *tmem38b^-/-^* mutant zebrafish Trap+ cells number was reduced compared to WT in presence of normal Rankl/Opg ratio resembling human and murine data (8, 16) and supporting a direct effect of Tric-b on osteoclast activity.

Of particular interest, in *tmem38b^Δ120-7/Δ120-7^* Trap+ cell number was unchanged compared to control, but their distribution was more abundant at the amputation site and along the neo-synthesized ray segment.

TRAP is an iron-containing enzyme expressed both in bone resorbing cells and in macrophages, that are one of the source of OCs (64). The increased expression of the macrophage marker *mpeg1* in *tmem38b^Δ120-7/Δ120-7^* compared to WT suggested that the Trap staining could indeed be due to immature OCs, possibly associated to a low level of anyway active mutant Tric-b (65).

### 4.6 Tric-b is necessary for zebrafish fibroblast and osteoblast homeostasis

Collagen type I extracted from bone and skin of both *tmem38b* zebrafish mutants showed a slightly faster electrophoretic migration resembling the pattern described for collagen type I synthesized by OI type XIV human fibroblasts and osteoblasts, for which a reduced lysine hydroxylation in the triple helical domain was demonstrated (16, 17). Thus, at a cellular level homozygosity for both mutant alleles affects collagen synthesis suggesting a need for a certain threshold of Tric-b conductance to guarantee proper cell homeostasis and that the mutant *in frame* protein likely conserves only a limited activity, sufficient for bone maintenance, but not enough for cell functionality. In human and murine OI *tmem38b* knock-out cells the collagen was mainly intracellularly retained causing severe matrix insufficiency. Interestingly, collagen extracellular deposition was also impaired in both zebrafish mutants in which reduced actinotrichia length was evident upon Picro Sirius red staining. A relevant role of lysine hydroxylase 1 in actinotrichia collagen post-translational modification was demonstrated in zebrafish using a knock down morpholino approach. Indeed, *lh1* morphants showed a dorsal curled tail phenotype, no actinotrichia development and defective formation of the fin fold (27). Since calcium regulates several post translational modifiers enzymes, including LH1, the correlation between defects in *TMEM38B* and impaired collagen folding already proposed by Cabral et al. (17) seems to hold up also in teleosts.

The mutant collagen retained inside the cells is responsible of enlarged ER cisternae in both zebrafish mutants and OI human and mouse cells. In *tmem38b^-/-^* and *tmem38b^Δ120-7/Δ120-7^* the upregulation of Hsp47 confirms impaired collagen secretion. Importantly, using the zebrafish models we demonstrated that the treatment with 4PBA partially rescues Hsp47 overexpression supporting its possible role as OI treatment also for OI type XIV as already demonstrated both *in vitro* and *in vivo* for dominant and some recessive OI forms (23, 35).

## Data availability statement

The original contributions presented in the study are included in the article/**Supplementary Material**. Further inquiries can be directed to the corresponding author.

## Ethics statement

The animal study was reviewed and approved by Italian Ministery of Health.

## Author contributions

Conceptualization: AF, FT, LL, VD. Methodology: AF, FT, LL, VD, RG, SC, IAKF, DL, SV. Validation: FT, LL, VD, SC, IAKF. Formal analysis: AF, FT,VD, PEW, AW, PC. Resources: AF. Data curation: AF, FT, VD. Writing - original draft: AF, FT, VD, LL. Writing - review and editing: AF, FT, VD, LL, SC, IAKF, AW, PC, BB, RB, SV, AR. Supervision: AF. Project administration: AF. Funding acquisition: AF. All authors contributed to the article and approved the submitted version.
