# Supplementary material for: Zebrafish Tric-b is required for skeletal development and bone cells differentiation
Source: Front Endocrinol (Lausanne). 2023 Jan 23;14:1002914. doi: 10.3389/fendo.2023.1002914 (PMC9899828; doi:10.3389/fendo.2023.1002914)
Supplement: Supplementary file 1 [file DataSheet_1.pdf]

Supplementary Material

1 Supplementary Materials and Methods

1.1 Generation of zebrafish *tmem38b* knock out mutants using CRISPR/Cas9

A single guide RNA (gRNA) for the *tmem38b* gene (ENSDARG00000100549, Ensemble 94) was designed using the online freely available software CHOPCHOP (https://chopchop.rc.fas.harvard.edu). The target sequence was selected at the 5' end, in exon 7 (5'- GGTTCTCGTCACTTCCTTCATGG - 3', 11391-11413 nt). The synthesis of target oligonucleotides (Eurofins Genomics), and the preparation of gRNA and Cas9 mRNA were carried out as described in (1). The genomic region surrounding the target sequence was PCR amplified using the following primers: forward 5'-TTACTGTCCGCTGGATGTGG-3' (11326-11345 nt) and reverse 5'–CAGAGCGTCGCTGTATTTGC-3' (11448-11467 nt), with annealing temperature of 56°C, generating a 142 bp amplicon. T7 endonuclease assay was performed to evaluate targeting efficiency as described in (1). The mutations in the heterozygous F1 zebrafish were determined by DNA extraction from tail clip of adult zebrafish followed by Sanger sequencing of the region surrounding the targeting.

To genotype *tmem38b*$^{-/-}$ and *tmem38b*$^{\Delta 120\text{-}7/120\text{-}7}$ mutants, amplicons were checked on 12% and 10% v/v electrophoresis acrylamide gel in TBE buffer (Tris HCl 0.1M, H_3BO_3 0.1M, EDTA 2mM, pH 8.2), respectively. The embryos generated from pairwise breeding were grown to 3 weeks post fertilization (wpf) and the genotyping data were used for Mendelian analysis of surviving homozygous knock out compared to WT and heterozygous zebrafish. Under the null hypothesis of no viability selection, progeny genotypes should conform to an expected Mendelian ratio of 1:2:1. Deviations from expected number of homozygous knock out were tested with goodness- of-fit Chi-square statistical analysis.

1.2 Cartilage staining

Cartilage were stained with Alcian Blue as described in Gioia et al. (2). Briefly, WT and *tmem38b*$^{-/-}$ zebrafish (n = 12 for each genotype) were fixed overnight in 4% (w/v) paraformaldehyde (PFA, Merck KGaA) at 4°C and stained in 0.02% (w/v) Alcian Blue (Sigma-Aldrich). Ventral images were acquired using M165 FC stereomicroscope (Leica) connected to DFC425C digital camera (Leica). The presence of cartilage deformities was investigated by measuring the angle of the ceratohyal (CH) cartilage (3). Measurements were performed using LAS v4.13 (Leica).

1.3 Bone Formation rate

Bone formation rate was performed on WT and *tmem38b*$^{-/-}$. Briefly, WT (n=9) and *tmem38b* $^{-/-}$ (n= 10) were stained with 0.01% Alizarin Red S (Sigma-Aldrich) in KOH, pH 7.4, from 6 to 10 dpf for 15 min every day, according to Bensimon-Brito et al 2016 (4). The fish were then stained in 0.2% calcein (Sigma-Aldrich) in 0.9% NaCl at 1 mpf for 10 minutes and then washed over night. Images were acquired using a Leica M165 FC microscope connected to Leica DFC425 C digital camera. Bone

formation rate was measured as the ratio between red operculum area on green operculum area, normalized to the standard length.

1.4 uCT

WT (n = 4) and *tmem38b*$^{-/-}$ (n = 4) zebrafish (9 mpf) were analyzed by µCT. Zebrafish were sacrificed, fixed overnight at 4 °C in 4% (w/v) PFA. Zebrafish were kept hydrated in parafilm and placed in a sample holder during µCT acquisitions (Skyscan 1272). For high-resolution scans and quantitative analysis of the 2nd and 3rd precaudal vertebrae, zebrafish were scanned at 40 kV and 230 mA with a voxel size of 3 µm. For all samples, ring artifact and beam hardening correction was kept constant and no smoothing was applied during reconstruction (NRecon, Bruker). After applying a constant global threshold to all samples, the morphological properties: vertebral body length (VBL, µm), bone volume (BV, mm^3), polar moment of inertia (MMIp, surrogate measure for resistance against torsion, mm^4), eccentricity, centrum thickness (C.Th, mm) and bone perimeter (B.Pm, mm) were analyzed according to previously established protocols (5, 6).

1.4 Nanoindentation

Elastic modulus E and hardness H of vertebral bone were determined using nanoindentation (iMicro, Nanomechanics, Inc., Oak Ridge, USA) based on previously established protocols (5). Whole fish samples (WT: n = 4; *tmem38b*$^{-/-}$ and *tmem38b*$^{\Delta 120\text{-}7/120\text{-}7}$ mutants n = 6, 2 mpf) were fixed in 4% PFA, embedded in polymethylmethacrylate, and ground plane parallel until the central sagittal plane was reached. To eliminate surface roughness, blocks were then polished with a 3 mm diamond suspension, followed by a 1 mm diamond suspension and final polishing with a 0.05 mm aluminum-oxide suspension. Finally, samples were ultrasonically cleaned in deionized water to remove surface debris. Using a Berkovich diamond tip and the depth-sensing continuous stiffness mode with a final depth of 1000 nm, indents were placed on the longitudinal plane of the vertebral bone cortex of 4 vertebrae per sample. Applying a Poisson's ratio of 0.3, E and H were extracted according to the Oliver-Pharr method (7) using in-house software (inView, Nanomechanics, Inc., Oak Ridge, USA).

2 Supplementary Results

2.1 Generation of *tmem38b* zebrafish models

A specific RNA guide (gRNA) targeting exon 7 of *tmem38b* was microinjected in 1-2 cell stage zebrafish fertilized embryos together with *in vitro* transcribed Cas9 mRNA. F0 mosaic zebrafish were screened for specific targeting at 1 day post fertilization (dpf) by T7 endonuclease I (T7EI) assay and by Sanger sequencing. The mutagenesis rate was 63%. To identify the F0 germ line zebrafish, mosaic fish were further outcrossed to AB WT. F1 progeny was initially screened by T7 endonuclease assay to discriminate the WT from the heterozygous mutant animals and the mutants finally confirmed by Sanger sequencing. The mutant F1 zebrafish carrying the c.524_530delTGAAGGA, predicted to insert a premature stop codon at amino acid 122 of Tric-b was chosen to obtain the F2 *tmem38b* knock out model (*tmem38b*$^{-/-}$). The mutant F1 zebrafish carrying the c.517_540del24nt, predicted to introduce the *in frame* p.Ala120_Thr127 deletion was selected to generate the F2 *tmem38b*$^{\Delta 120\text{-}7/\Delta 120\text{-}7}$. To genotype both *tmem38b*$^{-/-}$ and *tmem38b* $^{\Delta 120\text{-}7/\Delta 120\text{-}7}$ different size of PCR products were evaluated; the expected amplicons were 142 bp for the WT and 135 bp and 118 bp for *tmem38b*$^{-/-}$ and *tmem38b* $^{\Delta 120\text{-}7/\Delta 120\text{-}7}$, respectively (**Supplementary Figure 1B-D)**.

3 Supplementary Figures and Tables

3.1 Supplementary Figures

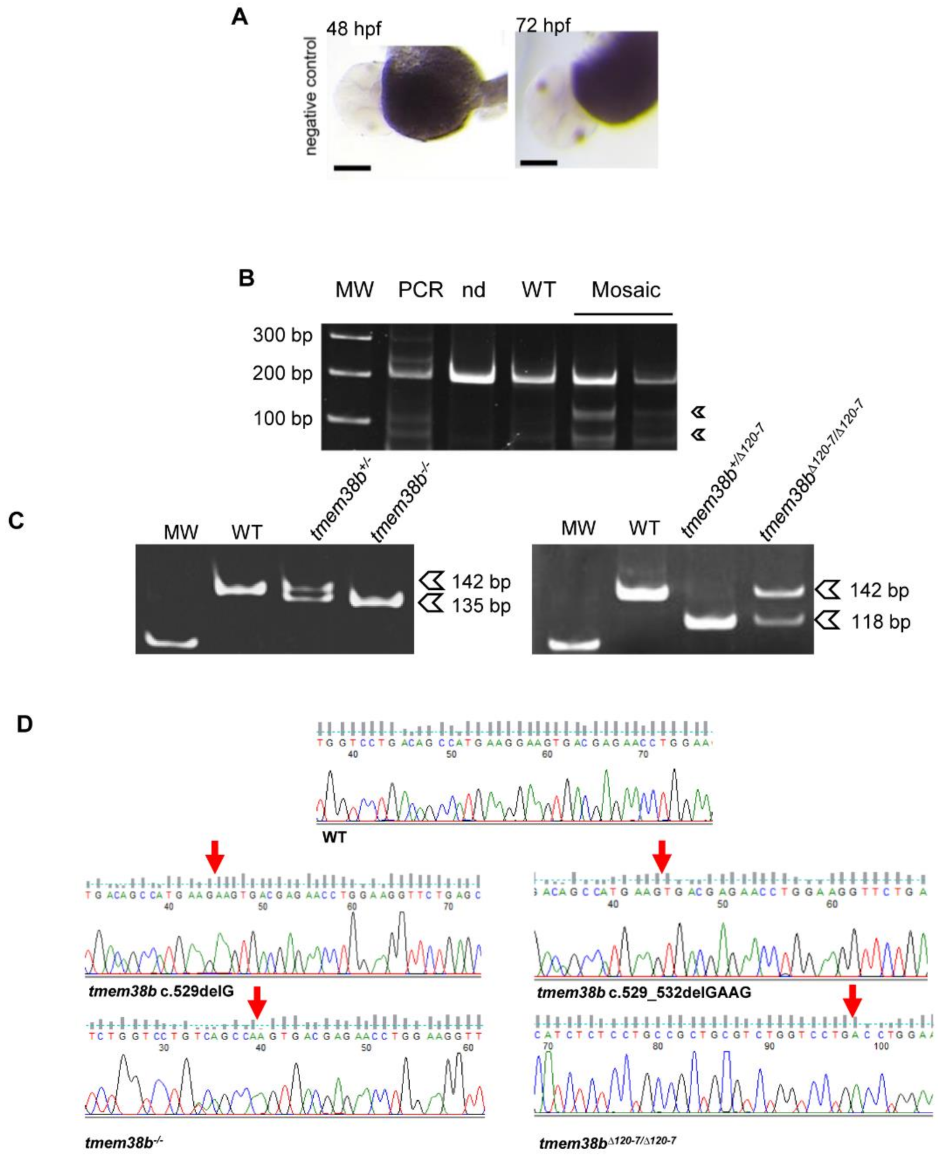

WT	5′-CTCCTGCCGCTGCGACTGGTCCTGACAGCCATGAAGGAAGTGACGAGAACCTGGAAGGTTCTGAGCGGCG-3′
tmem38b c.529delG p.*Glu123Lysfs*124*	5′-CTCCTGCCGCTGCGACTGGTCCTGACAGCCATGAAG-AAGTGACGAGAACCTGGAAGGTTCTGAGCGGCG-3′
tmem38b c.529_532delGAAG p.Glu123*	5′-CTCCTGCCGCTGCGACTGGTCCTGACAGCCATGAAG----TGACGAGAACCTGGAAGGTTCTGAGCGGCG-3′
tmem38b$^{-/-}$	5′-CTCCTGCCGCTGCGTCTGGTCCTGACAGCCA-------AGTGACGAGAACCTGGAAGGTTCTGAGCGGCG-3′
tmem38b$^{\Delta 120\text{-}7/\Delta 120\text{-}7}$	5′-CTCCTGCCGCTGCGTCTGGTCCTG------------------------ACCTGGAAGGTTCTGAGCGGCG-3′

Supplementary Figure 1. (A) *In situ* hybridization performed on 48 and 72 hpf WT embryos using the sense oligonucleotide as negative control. Scale bar: 500 µm. Generation of *tmem38b* zebrafish models using CRISPR/Cas9: **(B)** F0 mosaic fish screening using T7 endonuclease. Amplicons were digested by T7 endonuclease in presence of heterozygosity. MW: molecular weight, nd: not digested. **(C)** PCR analysis to genotype *tmem38b*$^{-/-}$ (left) and *tmem38b*$^{Δ120-7/Δ120-7}$ (right) mutants. Mutations in *tmem38b*$^{-/-}$ and *tmem38b*$^{Δ120-7/Δ120-7}$ could be distinguished by amplicon size. WT: 142 bp, *tmem38b*$^{-/-}$: 135 bp and *tmem38b*$^{Δ120-7/Δ120-7}$: 118 bp. MW: molecular weight. **(D)** DNA sequences of heterozygous mutations identified in F1 fish population. Red arrows and red dashes indicate nucleotide deletion regions.

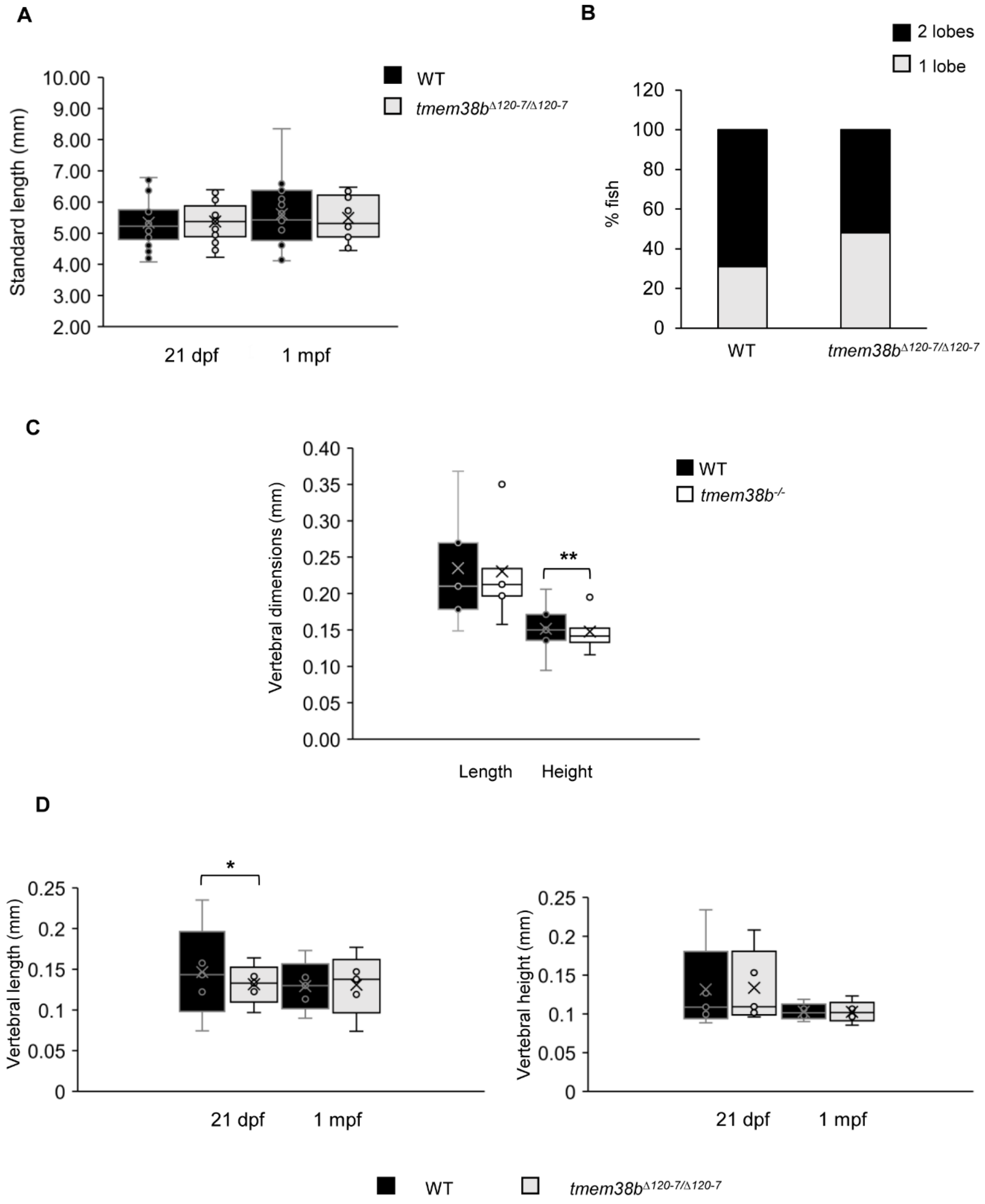
A
Standard length (mm)
10.00
9.00
8.00
7.00
6.00
5.00
4.00
3.00
2.00
21 dpf
1 mpf
WT
tmem38b$^{\Delta 120\text{-}7/\Delta 120\text{-}7}$
B
2 lobes
1 lobe
% fish
120
100
80
60
40
20
0
WT
tmem38b$^{\Delta 120\text{-}7/\Delta 120\text{-}7}$
C
Vertebral dimensions (mm)
0.40
0.35
0.30
0.25
0.20
0.15
0.10
0.05
0.00
Length
Height
**
WT
tmem38b$^{-/-}$
D
Vertebral length (mm)
0.25
0.2
0.15
0.1
0.05
0
*
21 dpf
1 mpf
Vertebral height (mm)
0.25
0.2
0.15
0.1
0.05
0
21 dpf
1 mpf
WT
tmem38b$^{\Delta 120\text{-}7/\Delta 120\text{-}7}$

Supplementary Figure 2. Morphometric analysis of WT and *tmem38b*$^{Δ120-7/Δ120-7}$. **(A)** Standard length of WT and *tmem38b*$^{Δ120-7/Δ120-7}$ measured at 21 dpf and 1 mpf. No difference was detected. 21 dpf: WT n=28, *tmem38b*$^{Δ120-7/Δ120-7}$ n=23; 1 mpf: WT n= 21, *tmem38b*$^{Δ120-7/Δ120-7}$ n = 13). **(B)** Swim bladder inflation levels was not impaired in *tmem38b*$^{Δ120-7/Δ120}$ respect to WT at 21 dpf (WT n = 26, *tmem38b*$^{Δ120-7/Δ120-7}$ n = 21). **(C)** Vertebral dimensions measured in 4 mpf WT and *tmem38b*$^{-/-}$(WT n = 15, *tmem38b*$^{--/}$ n = 11). While the vertebral length did not differ between WT and mutants, the vertebral height was reduced in *tmem38b*$^{-/-}$ respect to WT. **: $p < 0.01$. **(D)** Vertebral length was reduced in *tmem38b*$^{Δ120-7/Δ120-7}$ compared to WT at 21 dpf. *tmem38b*$^{Δ120-7/Δ120-7}$ showed no difference in vertebral height compared to WT. 21 dpf: WT n = 24, *tmem38b*$^{Δ120-7/Δ120-7}$ n = 19; 1 mpf: WT n = 18, *tmem38b*$^{Δ120-7/Δ120-7}$ n = 18.

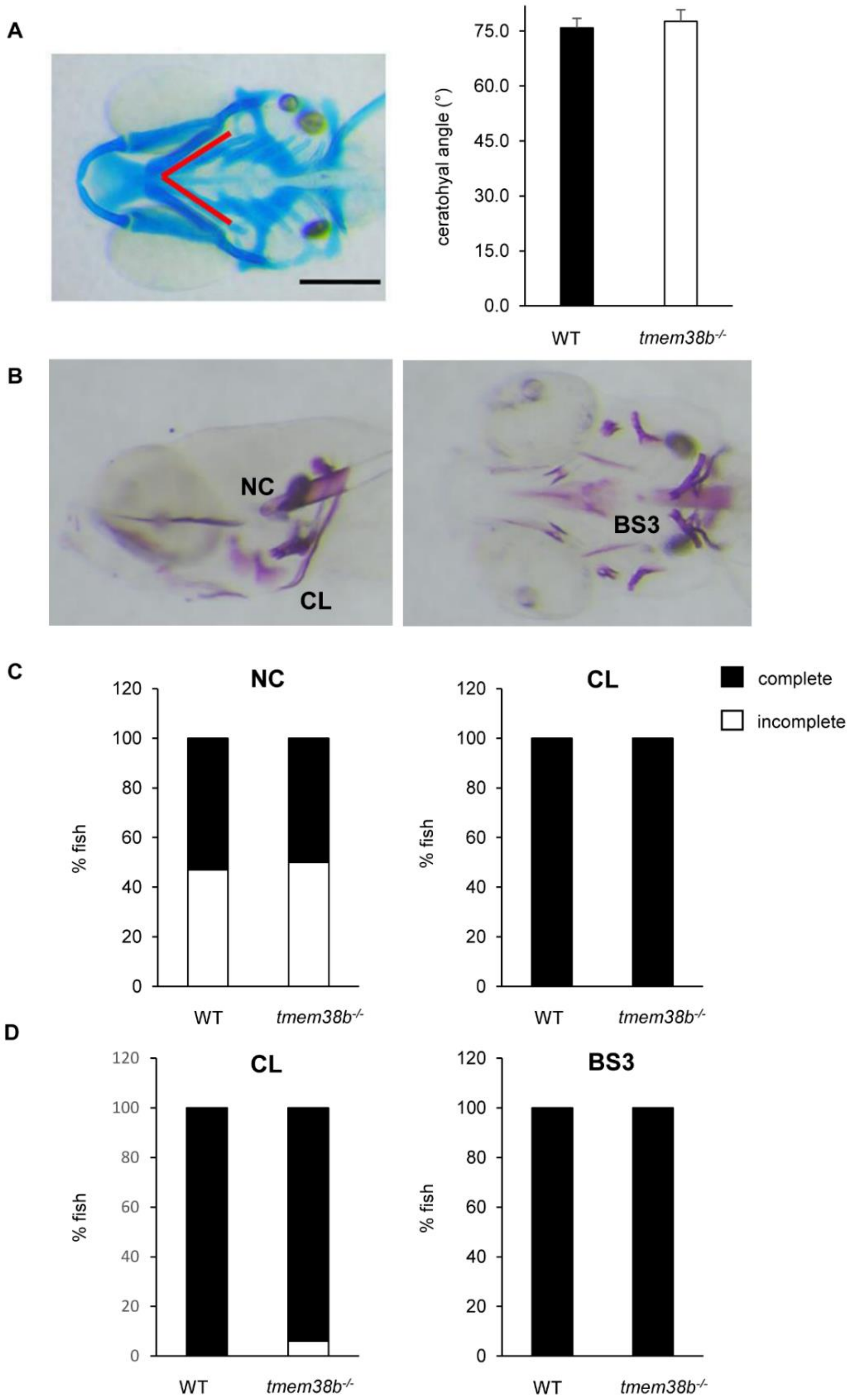

Supplementary Figure 3. *Tmem38b*$^{-/-}$ early skeletal development. **(A)** Representative image of zebrafish cranial cartilage stained with alcian blue (left). At 5dpf no difference in ceratohyal angle (red lines) was observed in *tmem38b*$^{-/-}$ with respect to WT ($n \geq 12$) . **(B)** Representative image of zebrafish cranial bones stained with alizarin red (CL:cleithrum, NC: notochord, BS3: branchiostegal ray 3). **(C)** At 7 dpf no difference in bone mineralization was observed in *tmem38b*$^{-/-}$ with respect to WT ($n \geq 18$). **(D)** At 14 dpf no difference in bone mineralization was observed in *tmem38b*$^{-/-}$ with respect to WT.

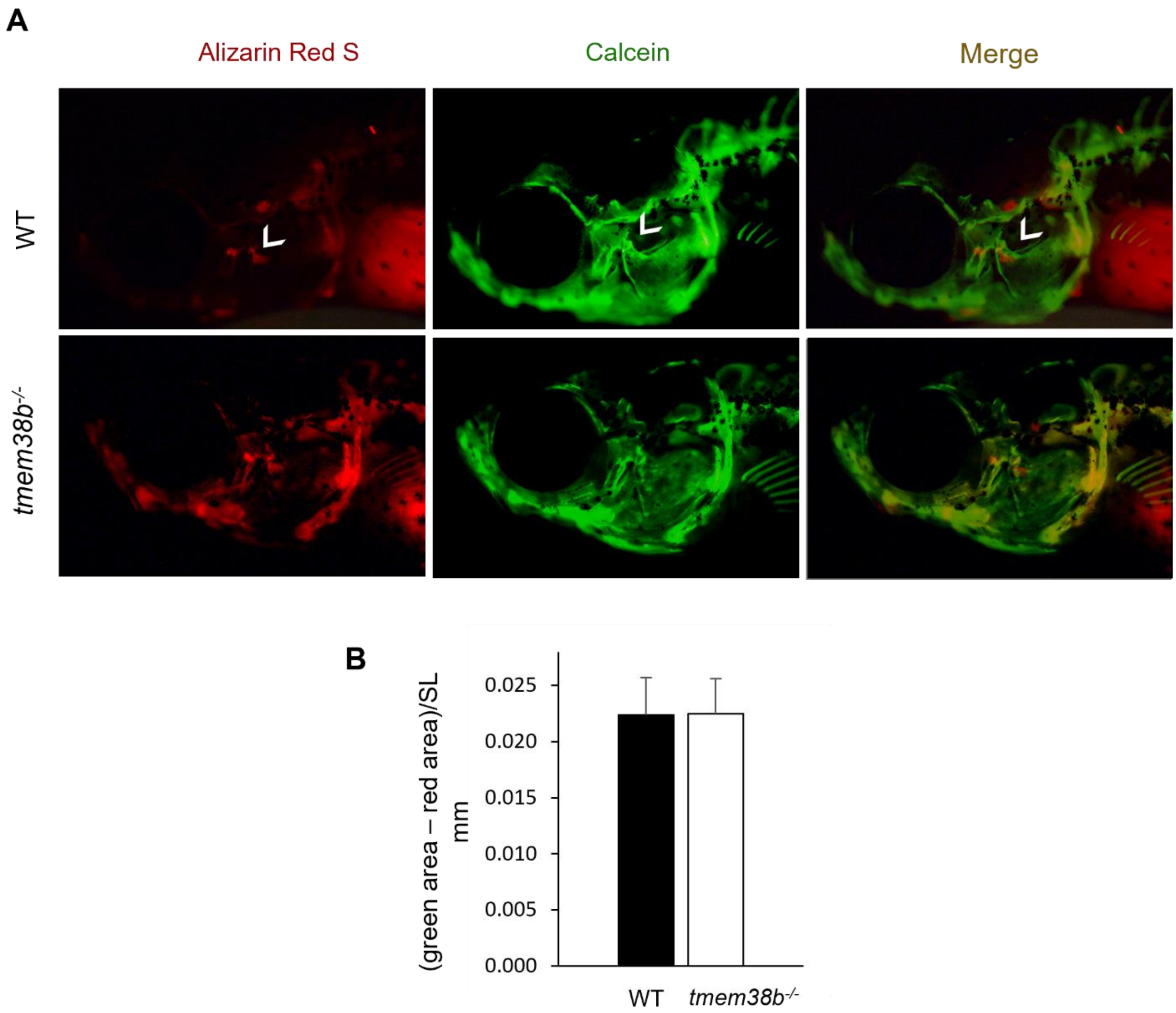

Supplementary Figure 4. Bone formation rate. **(A)** Representative images of WT and *tmem38b*$^{-/-}$ stained with Alizarin Red S from 6 to 10 dpf and with calcein at 1 mpf and merged image. **(B)** Bone formation rate, measured as the difference between red operculum area on green operculum area and normalized to standard length (SL), did not show difference between WT and *tmem38b*$^{-/-}$. White arrowheads indicate the operculum. Magnification: 5X.

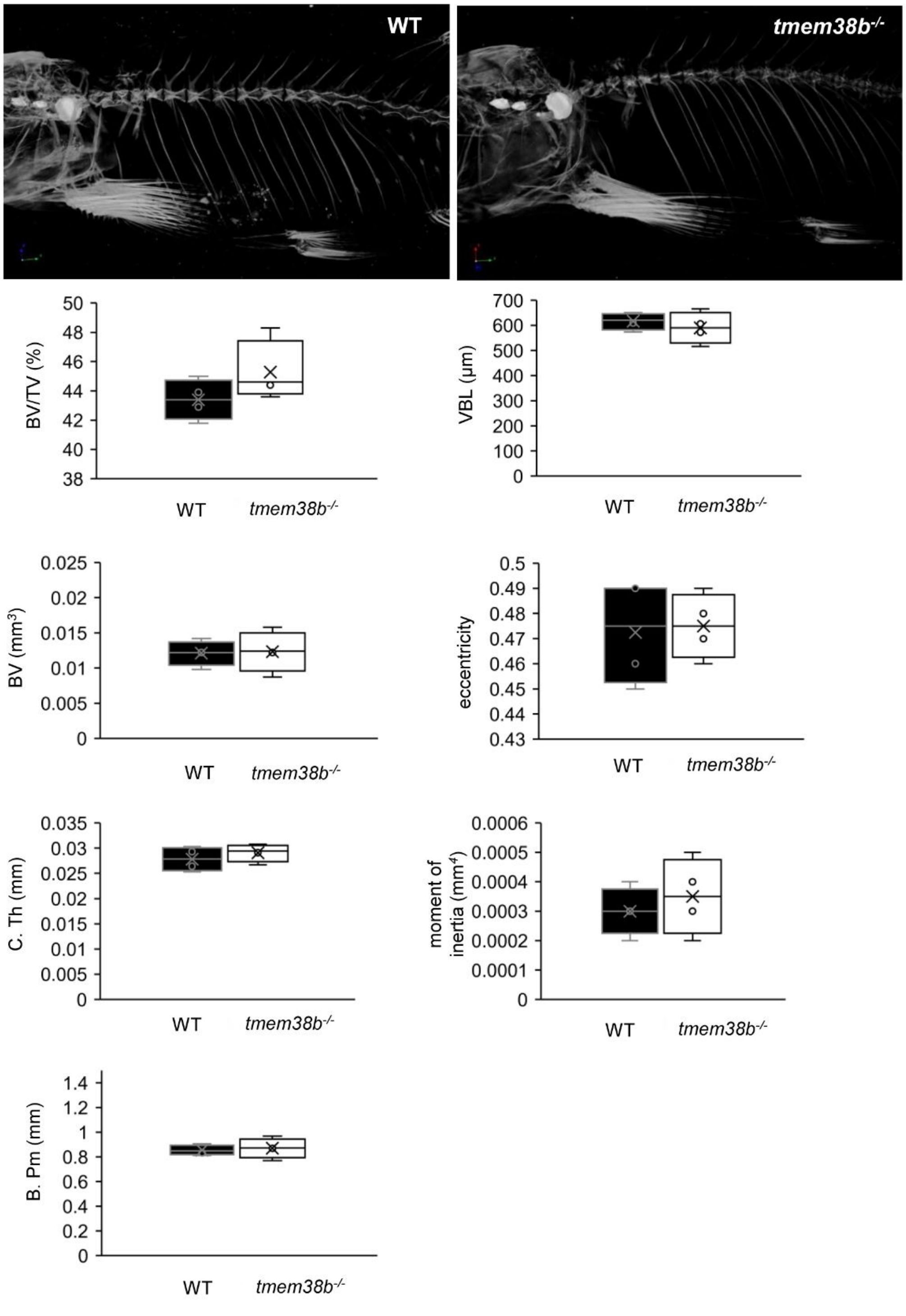

Supplementary Figure 5. µCT scans of adult WT (n = 4), and *tmem38b*$^{-/-}$ (n = 4). Morphometric analysis perfomed on the second and third precaudal vertebrae of each fish showed no difference between WT and *tmem38b*$^{-/-}$ for any of the parameters analyzed. BV/TV: bone volume on tissue volume, VBL: vertebral body length, BV: bone volume, C. Th: centrum thickness, B. Pm: bone perimeter.

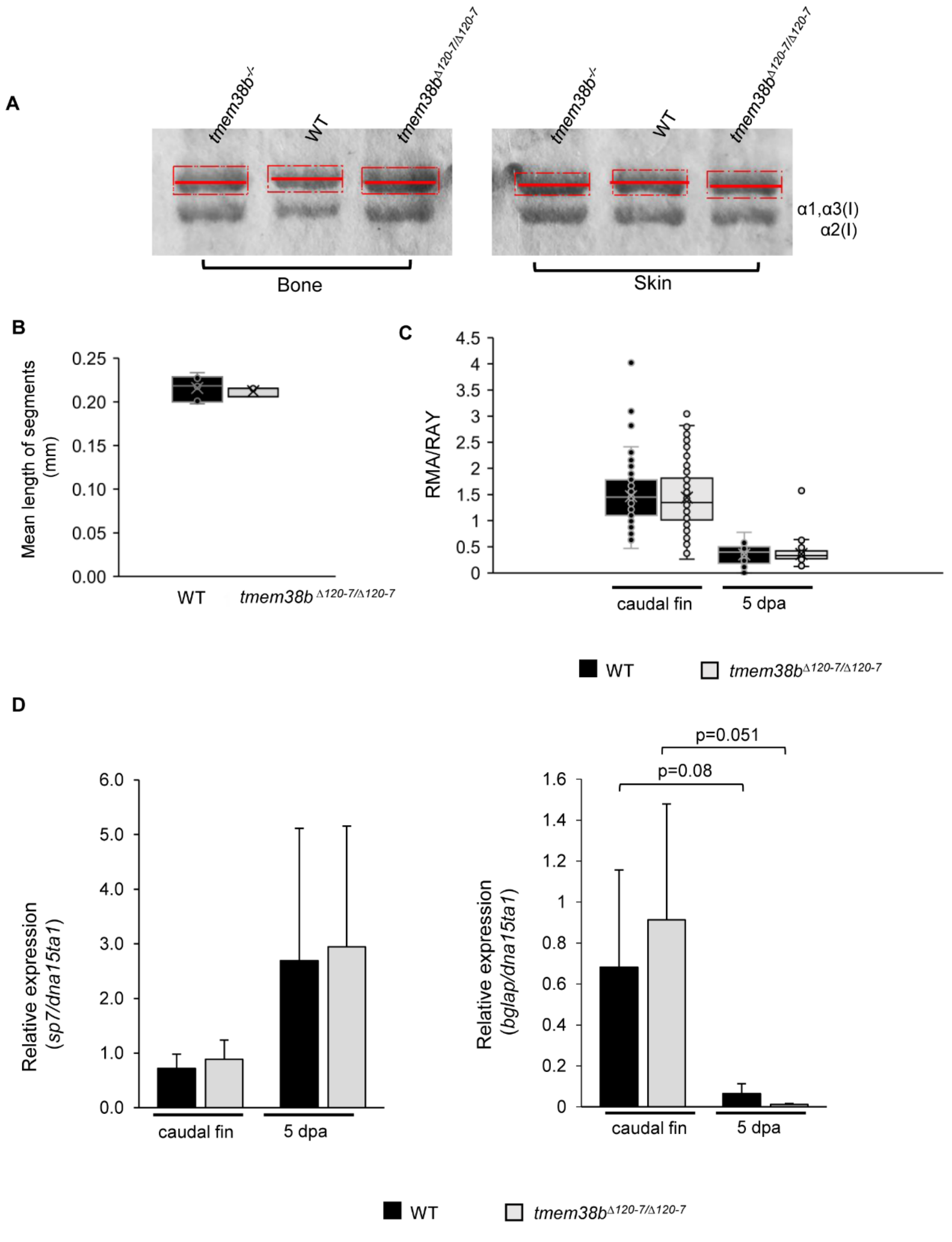
A
tmem38b-/-
WT
tmem38bΔ120-7/Δ120-7
tmem38b-/-
WT
tmem38bΔ120-7/Δ120-7
α1,α3(I)
α2(I)
Bone
Skin
B
Mean length of segments (mm)
0.25
0.20
0.15
0.10
0.05
0.00
WT
tmem38b Δ120-7/Δ120-7
C
RMA/RAY
4.5
4
3.5
3
2.5
2
1.5
1
0.5
0
caudal fin
5 dpa
WT
tmem38bΔ120-7/Δ120-7
D
Relative expression (sp7/dna15ta1)
6.0
5.0
4.0
3.0
2.0
1.0
0.0
caudal fin
5 dpa
p=0.051
p=0.08
Relative expression (bglap/dna15ta1)
1.6
1.4
1.2
1
0.8
0.6
0.4
0.2
0
caudal fin
5 dpa
WT
tmem38bΔ120-7/Δ120-7

Supplementary Figure 6. (A) Representative Coomassie stained SDS-Urea-PAGE of collagen type I extracted from WT and mutants (*tmem38b*$^{-/-}$; *tmem38b*$^{Δ120\text{-}7/Δ120\text{-}7}$) bone and skin. Mutants' collagen α bands are boxed in red. A red line crossing the middle of the box is used to underline the different migration. The mutant bands presented a slight faster migration compared to WT. Morphometric and bone mineralization analyses during caudal fin regeneration in WT and *tmem38b*$^{Δ120\text{-}7/Δ120\text{-}7}$ mutants: **(B)** The number and mean length of caudal fin ray segments were evaluated in the amputated tail. No difference was detected between WT and mutants for both measurements. **(C)** The ratio between the real mineralized area (RMA) and the mean ray width (RAY) was measured on alizarin red stained caudal fins to assess mineralization level. No difference in mineralization was found between WT and mutants in both the amputated and the 5 dpa samples (WT: $n \geq 9$, *tmem38b*$^{Δ120\text{-}7/Δ120\text{-}7}$ $n \geq 8$). **(D)** Relative expression of the early (*sp7*) and late (*bglap*) osteoblastic markers in WT and *tmem38b*$^{Δ120\text{-}7/Δ120\text{-}7}$ caudal fins in the amputated and 5 dpa samples (WT: $n = 3$, *tmem38b*$^{Δ120\text{-}7/Δ120\text{-}7}$ $n = 3$). No difference was detected in *sp7* and *bglap* expression between WT and *tmem38b*$^{Δ120\text{-}7/Δ120\text{-}7}$ at both time points. Data are expressed as mean ± SD.

3.2 Supplementary Tables

Supplementary Table 1. Genes flanking *TMEM38A* and *TMEM38B loci*

	Homo sapiens			***Mus musculus***			***Danio rerio***		
	**Gene name**	**Ensembl ID**	**Mb from *TMEM38A***	**Gene name**	**Ensembl ID**	**Mb from *Tmem38a***	**Gene name**	**Ensembl ID**	**Mb from *tmem38a***
	SMIM7	*ENSG00000214046*	-0,029	*Smim7*	*ENSMUSG00000044600*	-0,016	*smim7*	*ENSDARG00000074848*	-0,022
	NWD1	*ENSG00000188039*	0,127	*Nwd1*	*ENSMUSG00000048148*	0,13	*nwd1*	*ENSDARG00000076110*	0,067
	RAB3A	*ENSG00000105649*	1,51	*Rab3a*	*ENSMUSG00000031840*	-1,82	*rab3ab*	*ENSDARG00000043835*	-0,328
	PDE4C	*ENSG00000105650*	1,55	*Pde4c*	*ENSMUSG00000031842*	-1,83	*pde4cb*	*ENSDARG00000002411*	-0,385
	LSM4	*ENSG00000130520*	1,63	*Lsm4*	*ENSMUSG00000031848*	-1,90	*lsm4*	*ENSDARG00000023852*	-0,311
	KHLH26	*ENSG00000167487*	1,98	*Khlh26*	*ENSMUSG00000055707*	-2,11	*khlh26*	*ENSDARG00000053876*	-0,283
	CRTC1	*ENSG00000105662*	2	*Crtc1*	*ENSMUSG00000003575*	-2,14	*crtc1b*	*ENSDARG00000076076*	-0,221
	COMP	*ENSG00000105664*	2,1	*Comp*	*ENSMUSG00000031849*	-2,2	*comp*	*ENSDARG00000098431*	-0,205
	DDX49	*ENSG00000105671*	2,23	*Ddx49*	*ENSMUSG00000057788*	-2,28	*ddx49*	*ENSDARG00000012899*	-0,298

	Homo sapiens			***Mus musculus***			***Danio rerio***		
	**Gene name**	**Ensembl ID**	**Mb from *TMEM38B***	**Gene name**	**Ensembl ID**	**Mb from *Tmem38b***	**Gene name**	**Ensembl ID**	**Mb from *tmem38b***
	KLF4	*ENSG00000136826*	1,71	*Klf4*	*ENSMUSG00000003032*	1,67	*klf4*	*ENSDARG00000079922*	-0,153
	RAD23B	*ENSG00000119318*	1,55	*Rad23b*	*ENSMUSG00000028426*	1,56	*rad23b*	*ENSDARG00000021550*	-0,178
	ZNF462	*ENSG00000148143*	1,23	*Zfp462*	*ENSMUSG00000060206*	1,22	*znf462*	*ENSDARG00000112909*	-0,217

Supplementary Table 2. Percentage of surviving fish

**Age**	**WT**	***tmem38b***$^{+/-}$	***tmem38b***$^{-/-}$	**N**	***P* value**
**5 dpf**	27%	49%	24%	49	0.96
**7 dpf**	39%	45%	16%	49	0.06
**14 dpf**	32%	47%	21%	132	2.81E-33
**21 dpf**	32%	47%	22%	133	7.48E-34

N= number of fish, dpf= days post fertilization

References

1. Tonelli F, Cotti S, Leoni L, Besio R, Gioia R, Marchese L, et al. Crtap and p3h1 knock out zebrafish support defective collagen chaperoning as the cause of their osteogenesis imperfecta phenotype. Matrix Biol. 2020;90:40-60.

2. Gioia R, Tonelli F, Ceppi I, Biggiogera M, Leikin S, Fisher S, et al. The chaperone activity of 4PBA ameliorates the skeletal phenotype of Chihuahua, a zebrafish model for dominant osteogenesis imperfecta. Hum Mol Genet. 2017;26(15):2897-911.

3. Costantini A, Alm JJ, Tonelli F, Valta H, Huber C, Tran AN, et al. Novel RPL13 Variants and Variable Clinical Expressivity in a Human Ribosomopathy With Spondyloepimetaphyseal Dysplasia. J Bone Miner Res. 2021;36(2):283-97.

4. Bensimon-Brito A, Cardeira J, Dionísio G, Huysseune A, Cancela ML, Witten PE. Revisiting in vivo staining with alizarin red S--a valuable approach to analyse zebrafish skeletal mineralization during development and regeneration. BMC Dev Biol. 2016;16:2.

5. Fiedler IAK, Schmidt FN, Wölfel EM, Plumeyer C, Milovanovic P, Gioia R, et al. Severely Impaired Bone Material Quality in Chihuahua Zebrafish Resembles Classical Dominant Human Osteogenesis Imperfecta. J Bone Miner Res. 2018;33(8):1489-99.

6. Bouxsein ML, Boyd SK, Christiansen BA, Guldberg RE, Jepsen KJ, Müller R. Guidelines for assessment of bone microstructure in rodents using micro-computed tomography. J Bone Miner Res. 2010;25(7):1468-86.

7. Oliver WC, Pharr GM. An improved technique for determining hardness and elastic modulus using load and displacement sensing indentation experiments. Journal of Materials Research. 1992;7(6):1564-83.
